# Adhesion‐Driven Removal of Microplastics From Aquatic Systems by Using Microgel Glues

**DOI:** 10.1002/advs.75293

**Published:** 2026-04-14

**Authors:** Jinmeng Zhang, Jie Xu, Wen Chen, Yanping Chen, Zixin Pan, Weitai Wu

**Affiliations:** ^1^ State Key Laboratory For Physical Chemistry of Solid Surfaces Collaborative Innovation Center of Chemistry for Energy Materials The Key Laboratory For Chemical Biology of Fujian Province and Department of Chemistry College of Chemistry and Chemical Engineering Xiamen University Xiamen Fujian China

**Keywords:** adhesion‐driven aggregation, magnetic separation, microgel glues, microplastics removal, multivalent interactions

## Abstract

Microplastics (MPs) in aquatic ecosystems represent an escalating environmental challenge. Particularly, those with particle sizes below 1 µm exhibit strong Brownian motion and stable surface hydration layers, which hinder aggregation and render them exceptionally difficult to remove. Inspired by biological adhesion, we propose an adhesion‐driven co‐precipitation strategy that utilizes a soft polymeric microgel as a “glue” to aggregate dispersed MPs. Upon adhesion, MPs undergo directional transport toward solid‐liquid interfaces, where they accumulate into removable sediments. This adhesion‐driven interfacial deposition and co‐precipitation allows effective enrichment and physical separation of various types of microplastics, e.g., polystyrene (PS), polyethylene (PE), polypropylene (PP), and polyethylene terephthalate (PET). Remarkably, efficient removal (>90%) is retained even for nanoscale plastic particles as small as 50 nm. This strategy thus provides a broadly applicable and environmentally sustainable route for microplastic remediation in aquatic environments.

## Graphical abstract

Soft polymeric microgels function as “glues” that aggregate diverse microplastics through multivalent adhesion, promoting their interfacial deposition and co‐precipitation for efficient removal from water. Remarkably, this approach remains effective even for nanoscale plastic particles as small as 50 nm. Such interface‐driven adhesive capture offers a broadly applicable and environmentally conscious strategy for microplastic remediation in aquatic environments.

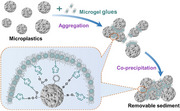

## 1 Introduction

**FIGURE 1:**
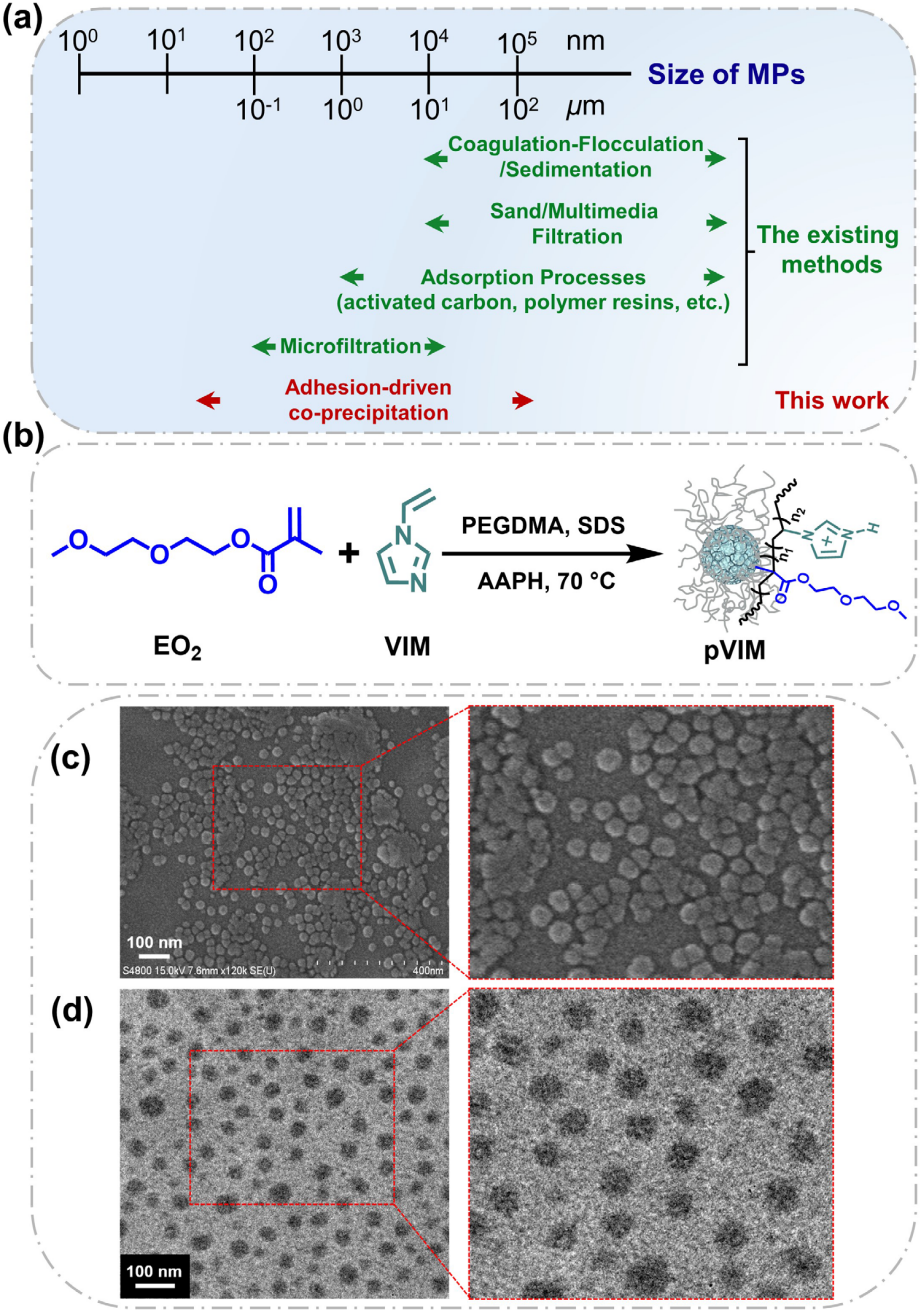
(a) Overview of microplastic size removal capabilities of conventional water treatment methods, and the adhesive‐driven co‐precipitation strategy developed in this work. (b) Synthesis of pVIM microgel glues for demonstrating the adhesion‐driven co‐precipitation strategy. (c, d) Scanning electron microscopy (SEM) and transmission electron microscopy (TEM) images of pVIM microgel glues.

The global plastics industry has grown rapidly since the 1960s, and annual production has now surpassed 400 million metric tons [1]. However, a significant portion of plastic waste is released into the natural environment rather than being effectively recycled and properly disposed of [2, 3, 4]. Plastic debris undergoes physical breakdown and chemical degradation, leading to the formation of microplastics (MPs), defined as plastic particles with a diameter <5 mm [5, 6, 7]. MPs have become pervasive contaminants in aquatic environments, posing a growing threat to ecological systems. The commonly used unit operations in water treatment processes, e.g., coagulation/flocculation, sedimentation, and sand filtration, cannot efficiently remove the microplastics with a particle size of 1–10 µm (Figure 1a) [8, 9, 10]. Notably, those plastics with even smaller particle sizes below 1 µm exhibit strong Brownian motion and stable surface hydration layers, which prevent their aggregation and make them particularly difficult to remove [11, 12, 13]. Consequently, this challenge highlights an urgent demand for effective strategies capable of directly removing submicrometer plastics from water.

In nature, organisms such as marine mussels, mayfly larvae, and carnivorous plants achieve strong underwater adhesion through multivalent, noncovalent interactions, enabling them to capture and aggregate microscopic objects even in dynamic aqueous environments [14, 15, 16, 17, 18, 19, 20]. Inspired by these biological mechanisms, a range of underwater adhesive systems, including acrylate resins, hydrogels, and nanostructured assemblies, has been explored to bind solid surfaces in wet environments [21, 22, 23, 24]. However, these materials often rely on bulk networks with limited interfacial adaptability and are not well‐suited for capturing small or irregular microplastic particles suspended in water. Building upon these insights, we hypothesized that soft and hydrated microgels will provide a different design concept because their deformable polymer networks can establish intimate contact and offer a high accessible surface area for efficient particle binding. Unlike conventional submerged acrylate‐based adhesives [25, 26, 27], the crosslinked microgels feature a highly swollen, nanoscale particulate architecture [28], enabling efficient adhesion‐driven aggregation of even submicrometer microplastics (<1 µm) while maintaining robust structural integrity under aqueous conditions.

As a proof of concept, we present a soft polymeric microgel that acts as a nanoscopic “glue”, inducing the aggregation of dispersed MPs. Polymeric microgels, consisting of crosslinked networks with high water content and tunable surface chemistry, provide an attractive platform to address the intrinsic challenges of underwater adhesion [29, 30, 31]. To realize this concept, we began by examining whether a structurally simple and compositionally conventional microgel could, through an adhesion‐driven mechanism, effectively address the long‐standing challenge of removing submicrometer microplastics. The microgel was engineered via copolymerization of 2‐(2‐methoxyethoxy)ethyl methacrylate (EO_2_) and vinyl imidazole (VIM). EO_2_ imparts hydrophilicity and chain mobility, generating a highly hydrated and flexible polymer network that facilitates conformal interfacial contact under aqueous conditions [32, 33, 34], while VIM introduces imidazole groups capable of reversible protonation and pH‐dependent charge regulation, thereby facilitating adaptive electrostatic interactions under environmentally relevant pH conditions. In addition, the hydrogen bonding capability of the imidazole moiety enhances interfacial affinity toward diverse polymer surfaces [35, 36, 37]. This molecular design allows the microgel to aggregate dispersed MPs through multivalent interactions, e.g., electrostatic attraction, hydrogen bonding, and hydrophobic association, ultimately achieving efficient removal of even nanoscale MPs.

## 2 Results and Discussion

The microgel glues (denoted as pVIM) were synthesized via free radical polymerization of 2‐(2‐methoxyethoxy)ethyl methacrylate (EO_2_) and 1‐vinyl imidazole (VIM), in the presence of poly(ethyleneglycol) dimethacrylate (PEGDMA) as a cross‐linker, 2,2’‐azobis(2‐methylpropionamidiine) dihydrochloride (AAPH) as an initiator, and sodium dodecyl sulfate (SDS) as a surfactant under N_2_ atmosphere at 70.0°C (Figure 1b). The synthesized polymer microgels exhibit a geoid‐like morphology in the dry state (Figure 1c,d). Using dynamic light scattering (DLS), the hydrodynamic size reached approximately 180 nm under an air atmosphere (Figure S1). In the Fourier transform infrared (FTIR) spectrum (Figure S2), a characteristic band of C─O─C stretching vibration (ca. 1121 cm^−1^) for the ether oxygen groups of PEGDMA unit, a characteristic band of O─C stretching vibration (1029 cm^−1^) for methyl ether oxygen on EO_2_ unit, and the vibration band at 1552 cm^−1^ and 1453 cm^−1^ correspond to the stretching vibrations of C═N and C─N of imidazole rings on VIM unit, respectively, confirming the introduction of the target monomer into the microgels. Additionally, X‐ray photoelectron spectroscopy (XPS) results show that N elements with binding energies of N1s were observed around 395 eV, which confirms the presence of imidazole groups on the microgel surface (Figure S3).

**FIGURE 2:**
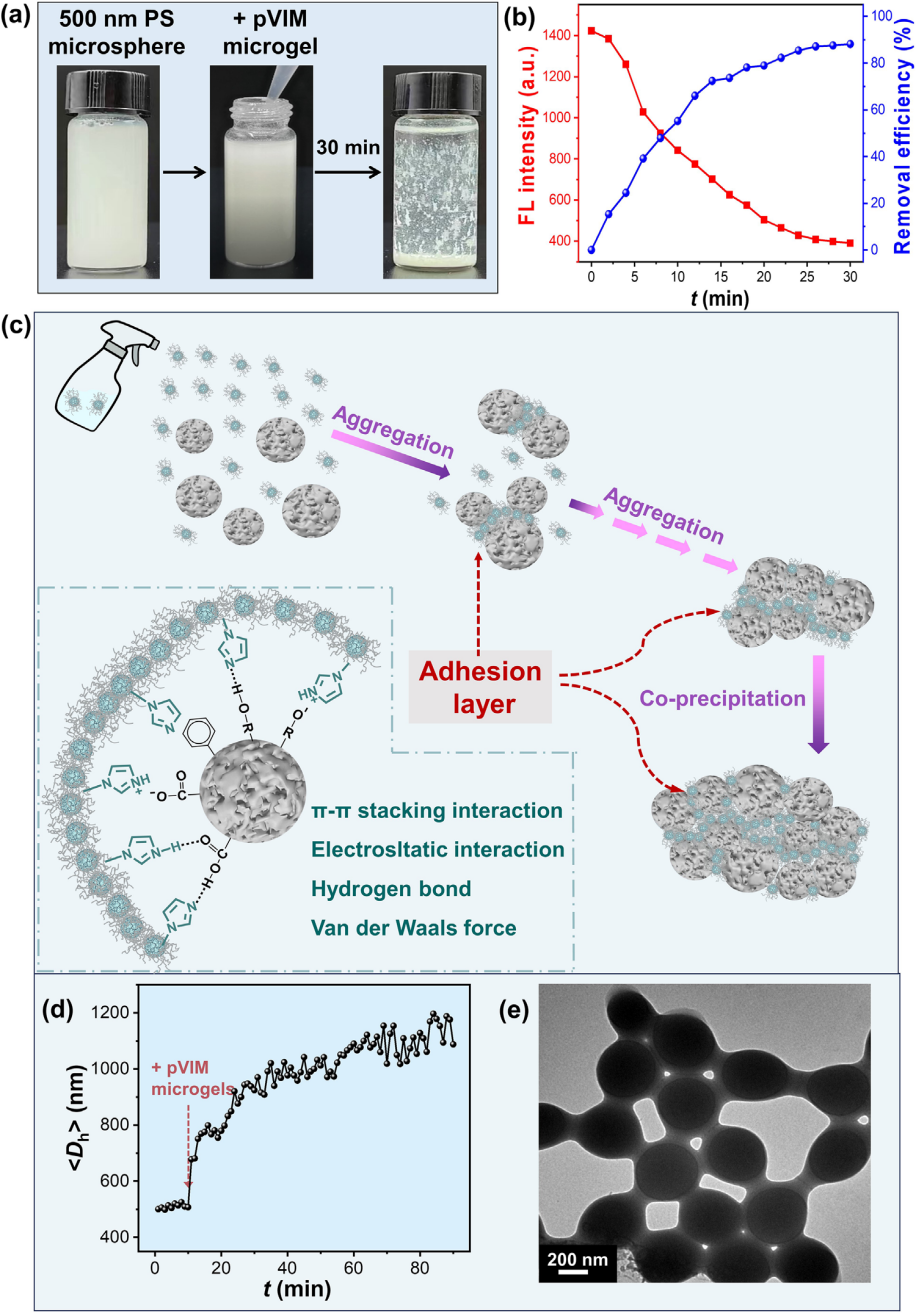
(a) Photographs of 1 mg·mL^−1^ of 500 nm PS fluorescent microspheres dispersed in water, after incubation with 0.2 mg·mL^−1^ pVIM for 30 min. (b) The changes of fluorescence intensity of the dispersion and the removal efficiency of PS microspheres over time when 1 mg·mL^−1^ 500 nm PS fluorescent microspheres were incubated with 0.2 mg·mL^−1^ pVIM for 30 min. (c) Schematic illustration of microplastics aggregated and co‐precipitated through adhesive‐driven by pVIM microgel glues. (d) Time‐dependent hydrodynamic size evolution of 500 nm PS microspheres (∼0.01 mg·mL^−1^) after the addition of pVIM microgel glues (∼0.01 mg·mL^−1^). (e) TEM image of PS microspheres (500 nm, 1.0 mg·mL^−1^) after incubation with pVIM microgel glues (0.2 mg·mL^−1^), showing a distinct low‐contrast adhesive layer surrounding the particle surface.

To elucidate the aggregation of MPs and interfacial interactions mediated by the designed microgels, polystyrene (PS) microspheres were selected as model MPs due to their wide use in daily products and their prevalence in aquatic plastic debris [38, 39, 40, 41]. PS MPs in wastewater typically carry a negative charge due to surface oxidation and adsorption of anionic organic matter [42, 43, 44]. In the absence of microgel glues, fluorescently labeled PS microspheres (Figures S4 and S5) remained uniformly dispersed in the suspension, exhibiting negligible affinity toward container walls. Upon the addition of pVIM microgel glues (0.2 mg·mL^−1^), a pronounced migration of MPs toward the solid‐liquid interface was observed, leading to the formation of densely packed wall‐bound sediments (Figure 2a; Movie S1). This process was corroborated by fluorescence intensity measurements of the supernatant, which exhibited a sharp decline within the first 30 min and subsequently reached a plateau. Quantitative analysis revealed a removal efficiency exceeding 90% (Figure 2b,c). The adhesion‐driven aggregation process was further verified by monitoring the temporal evolution of particle size using dynamic light scattering (DLS). Upon the introduction of a trace amount of pVIM microgel glues into an extremely dilute suspension of 500 nm PS microspheres, the effective hydrodynamic diameter increased by approximately 1200 nm within one hour (Figure 2d). This marked growth in particle size directly evidences that pVIM microgels induce the aggregation of PS microspheres. SEM images also revealed the presence of nanoscale gel particles firmly adhered to the surfaces of PS microspheres (Figure S6). TEM images displayed a low‐contrast interfacial layer coating the PS spheres, consistent with the formation of a thin microgel corona (Figure 2e; Figure S7), even for 50 nm PS microspheres (Figure S8). These observations indicate that the designed pVIM microgel glues effectively mediate the interfacial adhesion and aggregation of PS microspheres through the formation of a polymeric coating layer.

Recent studies have shown that underwater adhesion can be achieved through multiple noncovalent interactions, e.g., electrostatic attraction, hydrogen bonding, and others [45, 46, 47, 48]. To verify whether similar mechanisms govern our system, the elemental mapping analysis was first performed, and N and O signals were detected on the surface of PS microspheres. This observation suggests that the pVIM microgel glues (which contain N and O elements) have adhered to the PS microsphere surfaces (Figure S9). To further validate this inference, attenuated total reflectance Fourier transform infrared (ATR‐FTIR) spectroscopy and zeta potential measurements were performed. The ATR‐FTIR spectra revealed a broadened O─H/N─H stretching band (3200–3400 cm^−^^1^) and a red‐shifted C═O/C═N vibration (∼1730 cm^−^^1^), confirming the participation of hydrogen bonding and electrostatic interactions (Figure S10). Band broadening in the aromatic/imidazole region (1600–1500 cm^−^^1^) and overlapping peaks in the C─N/C─O─C region (1250–1050 cm^−^^1^) indicated π‐π interactions and close interfacial contact between the microgel and PS surfaces. Zeta potential measurements confirmed charge neutralization and interparticle attraction upon microgel addition (Figures S11 and S12). These results support the hypothesis that the adhesion and subsequent aggregation of MPs are primarily driven by multivalent, noncovalent interactions, establishing a molecular‐level basis for the adhesion‐induced co‐precipitation process.

Differential scanning calorimetry (DSC) analysis was performed to determine the glass transition temperature (T_g_) of the dried microgel. The T_g_ was measured to be −1.07°C (Figure S13), which is well below room temperature. This indicates that the low‐crosslink‐density polymer chains on the microgel surface [49, 50, 51] maintain favorable chain mobility and interfacial compliance during the adhesion tests, which facilitates efficient binding with microplastic particles. When the operating temperature is significantly above T_g_, polymer chain mobility is enhanced, allowing deformation and adaptive surface matching. In contrast, as temperature approaches T_g_, reduced segmental mobility could limit conformal contact and weaken adhesion. Given that the measured T_g_ is substantially lower than the experimental temperature (25°C), such limitations are unlikely under the present conditions.

**FIGURE 3:**
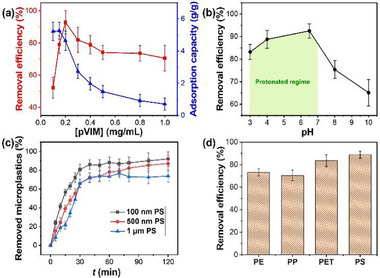
(a) Effect of pVIM microgel glue dosage on the removal efficiency and removal capacity for 500 nm PS MPs (1.0 mg·mL^−1^). (b) Effect of pH on the removal efficiency for 500 nm PS MPs (1.0 mg·mL^−1^). (c) Removal of the PS MPs of different particle sizes (100 nm, 500 nm, and 1 µm) by 0.2 mg·mL^−1^ pVIM microgel glues. (d) Removal efficiencies of different microplastic types (PE, PP, PET, PS; 50–100 µm; the MPs are representative of debris originating from the fragmentation of various commonly used plastic goods) by pVIM microgel glues.

To further verify that the aggregation and co‐sedimentation of MPs are driven by interfacial adhesion rather than nonspecific coagulation, we systematically investigated the influence of microgel concentration, pH, and ionic strength on MP removal. The removal efficiency initially increased with microgel concentration, reaching a maximum of 92.5% at 0.2 mg·mL^−1^ (Figure 3a). Further increases in concentration resulted in a gradual decline in performance, likely because excess microgels formed stable colloidal dispersions in water, which competed with MPs for surface interactions and hindered interfacial deposition efficiency [52]. The removal behavior exhibited a pronounced pH dependence consistent with the protonation and charge regulation of imidazole moieties within the pVIM network [36]. Maximum efficiency was achieved under mildly acidic to neutral conditions (pH 5–7) (Figure 3b), where optimal charge balance and hydrogen‐bonding strength facilitated adhesion. The system also maintained high performance under moderate ionic strengths (up to 100 mM NaCl), demonstrating the robustness of the noncovalent adhesion mechanism under environmentally relevant conditions (Figure S14). Importantly, the pVIM microgel glues exhibited remarkable capability in aggregating and removing submicrometer plastic particles. Owing to its large specific surface area and high dispersibility in aqueous media, efficient adhesion‐induced deposition was achieved for PS microspheres with diameters of 1 µm, 500 nm, and 100 nm, yielding removal efficiencies of 85.2%, 92.5%, and 93.1%, respectively (Figure 3c). These results not only validate that the observed aggregation and sedimentation are governed by adhesion‐driven interactions but also demonstrate the adaptability of this mechanism across diverse aqueous conditions.

To further elucidate the role of the imidazole functionality, microgel glues with identical total monomer content but varied EO_2_ to VIM ratios (1:0, 1:1, and 2:1) were synthesized. Increasing the VIM fraction produced larger and more positively charged microgels, with hydrodynamic sizes of 62.95 nm, 182.36 nm, and 247.90 nm and ζ‐potentials of −13.19 mV, 22.59 mV, and 30.8 mV for pEO_2_, pVIM‐1, and pVIM‐2, respectively. These structural changes led to systematic improvements in the removal of 50 nm PS nanoplastics, yielding efficiencies of 73.47%, 86.29%, and 91.38% (Figure S15). XPS analysis revealed a systematic increase in surface nitrogen content with increasing VIM fraction (Figure S16). A positive correlation between nitrogen content and nanoscale microplastic removal efficiency was observed, supporting the role of imidazole‐mediated interfacial interactions.

To evaluate the versatility and broad applicability of the pVIM microgel glues, we next examined their ability to aggregate various environmentally relevant MPs. PS, polyethylene (PE), polypropylene (PP), and polyethylene terephthalate (PET) microplastics (Figures S17–S19), which exhibit negligible fluorescence, were evaluated by gravimetrically measuring the residual mass of particles in the supernatant after microgel treatment and subsequent drying. Upon incubation with the microgel, all four types of MPs (50–100 µm) exhibited significant removal efficiencies of 88.74%, 73.12%, 70.35%, and 83.59%, respectively (Figure 3d). Although PE and PP do not contain aromatic groups and therefore cannot participate in π–π interactions, their mechanically fragmented particles exhibit negative zeta potentials, which enables electrostatic attraction with the positively charged imidazole groups in the microgel. Hydrophobic association between the aliphatic microplastic surfaces and the hydrophobic domains of the microgel, together with van der Waals forces, further assists the interfacial attachment (Figure S20). Highlighting the material's broad‐spectrum adhesion and separation capability.

**FIGURE 4:**
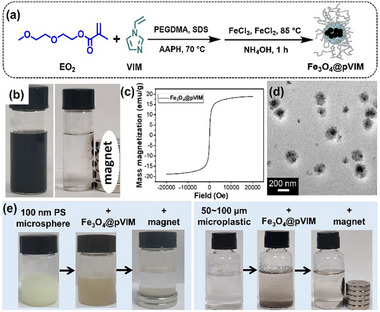
(a) Schematic illustration for the fabrication of Fe_3_O_4_@pVIM microgel glues. (b) Photographs of the Fe_3_O_4_@pVIM microgel glues before and after magnetic separation. (c) VSM magnetization curves of the Fe_3_O_4_@pVIM microgels. (d) Typical TEM image of the Fe_3_O_4_@pVIM microgel glues. (e) Photographs of 1 mg·mL^−1^ of 100 nm PS fluorescent microspheres and 50–100 µm microplastics dispersed in water, after incubation with 0.2 mg·mL^−1^ Fe_3_O_4_@pVIM, and after magnetic separation.

In practical implementation, trace amounts of residual pVIM microgel glues were detected in the treated effluent. To address this issue, Fe_3_O_4_ nanoparticles were incorporated in situ into the microgel network to introduce magnetic responsiveness while preserving the hydrated and compliant polymer architecture. The integration of magnetic domains enables rapid and efficient separation under an external magnetic field, thereby minimizing microgel residues and improving operational recoverability (Figure 4a,b). After magnetic collection, dynamic light scattering (DLS) analysis of the supernatant revealed no discernible scattering signal, further confirming that the integration of magnetic separation effectively eliminates microgel residues in the treated water. The synthesized Fe_3_O_4_@pVIM samples showed superparamagnetic property with a saturation magnetization of 18.8 emu/g from vibrating sample magnetometry (VSM, Figure 4c; Figure S21). The purified Fe_3_O_4_@pVIM microgel glue was characterized by using TEM, in which a gel layer with low contrast appears on the surface of aggregates of Fe_3_O_4_ nanoparticles, indicating a core‐shell structure (Figure 4d; Figures S22 and S23). Using dynamic light scattering (DLS), the hydrodynamic size reached approximately 1000 nm (Figure S24), suggesting aggregation. The crystalline structure of the synthesized Fe_3_O_4_@pVIM microgel glues was confirmed by XRD (Figure S25).

**FIGURE 5:**
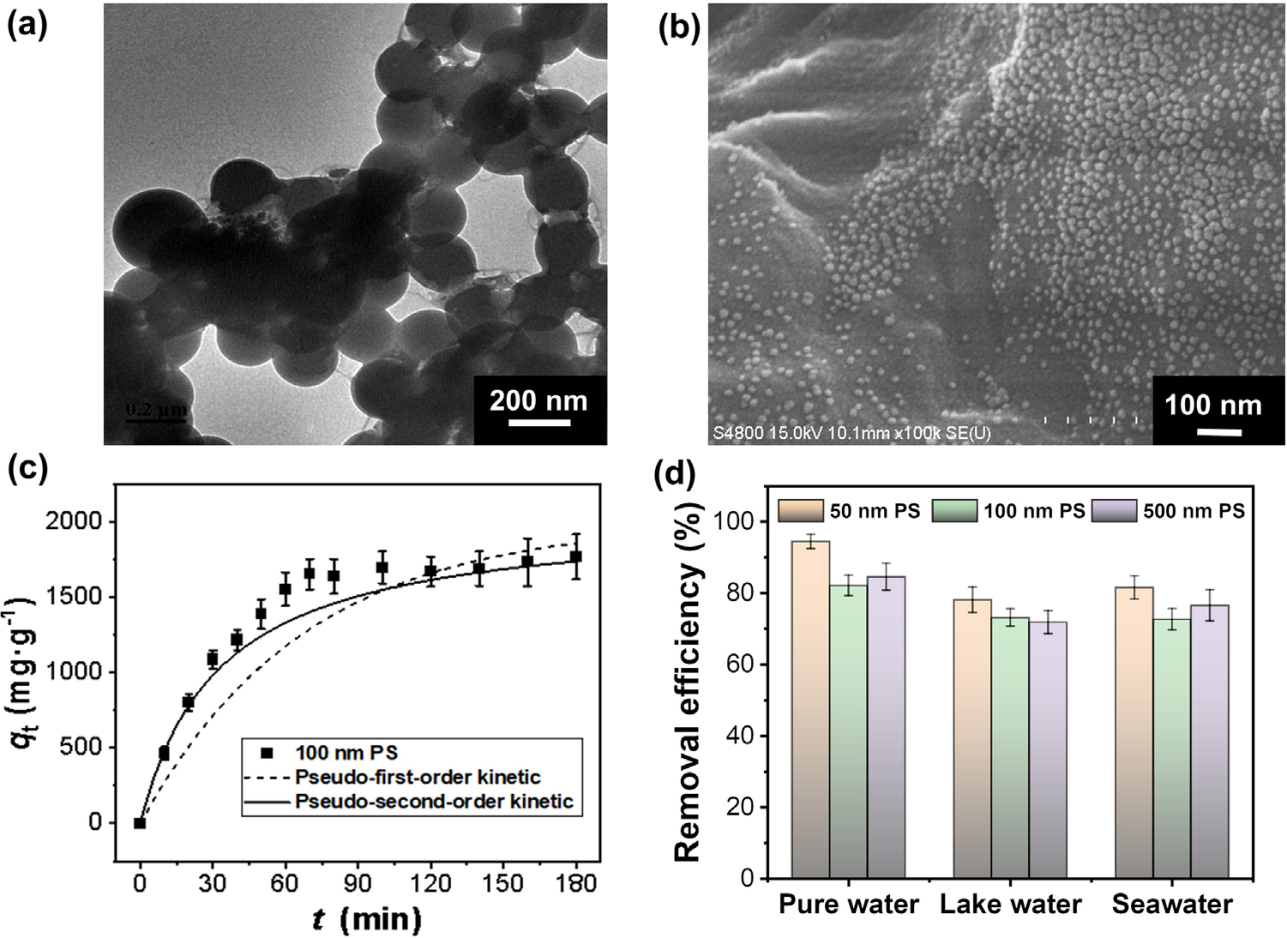
(a) A typical TEM image showing Fe_3_O_4_@pVIM adhered to the surface of PS microspheres. (b) A SEM image illustrating Fe_3_O_4_@pVIM anchored on the surface of 50–100 µm PS MPs. (c) Time‐dependent removal of 100 nm PS MPs by Fe_3_O_4_@pVIM microgel glues, fitted with pseudo‐first‐order and pseudo‐second‐order kinetic models. (d) Comparison of PS MP (50 nm, 100 nm, and 500 nm, 1 mg·mL^−^^1^) removal efficiencies by Fe_3_O_4_@pVIM in pure water, lake water, and seawater. Lake water was collected from Furong Lake at Xiamen University, and seawater was sampled from Baicheng Beach near Xiamen University (Xiamen, China).

To evaluate the size‐dependent removal efficiency of Fe_3_O_4_@pVIM microgel glues toward microplastics (MPs), fluorescent PS microspheres with diameters of 50–500 nm and large MPs ranging from 50–100 µm were employed as model pollutants (Figure 4e). Direct microscopic evidence supporting the adhesion‐driven aggregation mechanism was obtained through electron microscopy, which clearly revealed the interfacial association between Fe_3_O_4_@pVIM microgel glues and PS particles (Figures 5a,b). At an initial PS concentration of 1.0 mg·mL^−1^, a low microgel dosage of 0.2 mg·mL^−1^ achieved a removal efficiency exceeding 90% for 100 nm PS MPs, with a corresponding adsorption capacity reaching 1696.17 mg·g^−1^ (Figure 5c). The pseudo‐second order model exhibited a higher correlation coefficient, suggesting that the process is governed by interfacial interaction kinetics rather than simple diffusion control (Table S1). The removal efficiency further increased with mixing time. After 90 min of oscillation, magnetic separation yielded removal efficiencies of 86.3%, 72.5%, 79.4%, and 81.6% for PS, PE, PP, and PET MPs, respectively (Figure S26). Notably, the removal efficiency displayed an inverse dependence on particle size, with smaller MPs being more effectively aggregated. This phenomenon can be attributed to the initially limited sedimentation of nanoscale particles caused by high surface tension forces in water. With prolonged mixing, particle aggregation enlarged the effective hydrodynamic diameter, reducing interfacial resistance and thereby facilitating both sedimentation and subsequent magnetic retrieval. Microplastic removal did not reach 100% primarily due to particle heterogeneity in size, shape, and surface chemistry, which leads to variable interactions with the microgel [53]. Kinetic and steric constraints can also prevent some small or weakly interactive MPs from contacting the microgel in a single treatment. Sequential treatments may improve overall removal, but complete 100% clearance is inherently challenging. Nevertheless, the microgel efficiently captures the vast majority of MPs in one step, demonstrating its strong potential for practical remediation.

Compared to previously reported magnetic materials for microplastic remediation, the Fe_3_O_4_@pVIM microgels developed in this work achieved efficient removal of submicron PS MPs (≤1 µm) with a significantly lower dosage of magnetic nanoparticles (Table S2). Recent magnetic separation approaches based on composite magnetic particles or PEG‐modified Fe_3_O_4_ nanoparticles perform well for microplastics in the micrometer range, but their rigid inorganic cores or thin polymer coatings provide limited interfacial adaptability toward smaller or irregular particles [54, 55].

To assess whether the designed microgel glues can function as a regenerable adhesive system, we first examined their intrinsic CO_2_‐responsive behavior arising from the reversible protonation of imidazole groups. The hydrodynamic size, ζ‐potential, and pH of the pVIM microgels were monitored under alternating CO_2_ and N_2_ atmospheres (Figures S1 and S27). CO_2_ bubbling protonated the imidazole moieties, leading to an increase in positive surface charge, microgel swelling, and a decrease in dispersion pH due to carbonic acid formation. Subsequent N_2_ purging removed dissolved CO_2_, reversed protonation, and restored both the microgel size and pH, confirming the dynamic and reversible charge regulation inherent to the network [56, 57, 58]. Building on this stimulus responsiveness, we evaluated whether microplastic binding could be modulated through gas switching. A release efficiency of 37.8% was achieved for 100 nm PS particles, demonstrating that attachment and detachment can be controlled through mild CO_2_/N_2_ cycling (Figure S28). Coupled with the magnetic recoverability imparted by Fe_3_O_4_ nanoparticles, these results show that the microgel glues are readily regenerable rather than single‐use materials, offering a more sustainable and reusable platform for microplastic remediation.

To evaluate the practical applicability of Fe_3_O_4_@pVIM microgel glues, their performance was systematically examined in representative natural water samples, including seawater and lake water, with ultrapure water serving as a reference (Figure 5d). The physicochemical parameters of the collected natural waters were characterized to ensure reproducibility, including pH, turbidity, conductivity, and total dissolved solids (TDS) (Table S3). The lake water exhibited near‐neutral pH and moderate turbidity, whereas seawater displayed elevated conductivity and ionic strength as expected. Despite these compositional differences, the microgel glues maintained high and consistent removal efficiencies toward PS MPs of different sizes, including 50 nm, 100 nm, and 500 nm. Removal efficiencies exceeded 90% in ultrapure water and remained above 85% in both seawater and lake water. Although dissolved organic matter and background colloids present in natural waters may compete for interfacial binding sites or influence microplastic dispersion stability, the multivalent adhesion mechanism, which combines electrostatic attraction, hydrogen bonding, and hydrophobic association, remains effective under these environmentally relevant conditions. These results demonstrate the robustness and adaptability of the adhesive magnetic separation strategy in complex aqueous matrices.

## 3 Conclusion

In summary, we have developed a soft polymeric microgel system that functions as a nanoscale adhesive platform for the efficient removal of microplastics from aqueous environments. The hydrated and compliant network enables cooperative interfacial interactions, including electrostatic attraction, hydrogen bonding, and hydrophobic association, thereby promoting aggregation, interfacial deposition, and co‐precipitation of dispersed microplastics. The incorporation of Fe_3_O_4_ nanoparticles introduces magnetic responsiveness, enabling efficient external field‐driven retrieval and minimizing potential secondary contamination from residual microgels. The system achieves removal efficiencies exceeding 90% for both micrometer‐scale and nanoscale plastic particles down to 50 nm, a size regime that remains challenging for conventional water treatment processes. Furthermore, consistent performance in natural water matrices and reversible CO_2_ responsiveness demonstrate the adaptability and regenerability of the material. Future efforts may focus on optimizing scalable fabrication strategies and evaluating integration with existing wastewater treatment infrastructures. Systematic investigation of long term operational stability, continuous flow performance, and real wastewater complexity will further facilitate practical implementation.

## Author Contributions

Jinmeng Zhang and Jie Xu performed the experiment and wrote the manuscript. The rest of the authors participated in the design and discussion of the experiment and results. Weitai Wu directed the whole program. The manuscript was written through the contributions of all authors.

## Conflicts of Interest

The authors declare no conflict of interest.

## Supporting information




**Supporting File 1**: advs75293‐sup‐0001‐SuppMat.docx.

**Supporting File 2**: advs75293‐sup‐0002‐MovieS1.mp4.

## Data Availability

The data that support the findings of this study are available from the corresponding author upon reasonable request.
