# Supplementary material for: Adhesion‐Driven Removal of Microplastics From Aquatic Systems by Using Microgel Glues
Source: Adv Sci (Weinh). 2026 Apr 14;13(38):e75293. doi: 10.1002/advs.75293 (PMC13335070; doi:10.1002/advs.75293)
Supplement: Supplementary file 1 — Supporting File 1: advs75293‐sup‐0001‐SuppMat.docx. [file ADVS-13-e75293-s002.docx]

Adhesion-Driven Removal of Microplastics from Aquatic Systems by Using Microgel Glues

Jinmeng Zhang,^+^ Jie Xu,^+^ Wen Chen, Yanping Chen, Zixin Pan, and Weitai Wu *

[^+^] These authors contributed equally to this work.

[*] Corresponding authors.

**Table of Contents:**

**Experimental section**

Materials

Synthesis and Characterization of Microgels

**Tables**

**Table S1:** Kinetic parameters and correlation coefficients obtained from pseudo first order and pseudo second order fitting models for PS microspheres removal by Fe3O4@pVIM microgel glues.

**Table S2:** Comparison of microplastic removal efficiency between this work and a few literature reported methods.

**Table S3:** Physicochemical parameters of tested natural waters.

**Figures**

**Figure S1-S28**

**References for Supporting Information**

**Experimental Section**

**Materials**

(2-(2-Methoxyethoxy)ethoxy)ethyl methacrylate (EO_2_), 1-vinyl imidazole (VIM), poly(ethyleneglycol)dimethacrylate (PEGDMA), and 2′-azobis(2-methylpropionami-dine) dihydrochloride (AAPH) were purchased from Sigma Aldrich. Sodium dodecyl sulfate (SDS), and iron (III) chloride hexahydrate (FeCl_3_×6H_2_O), sodium chloride (NaCl), sodium hydroxide (NaOH), hydrochloric acid (HCl), and ammonium hydroxide (NH_4_OH) were purchased from Sinopharm Chemical Reagent Co., Ltd. Iron (II) chloride tetrahydrate (FeCl_2_×4H_2_O) was supplied by [Alfa Aesar](http://www.baidu.com/baidu.php?url=060000KKXYb9K48fS36dF-cNNDYRhrBRmU80oPapBelnKx7PDYEhN40y2a5RIVhnI6UyDgnDpghtDsKGYi27NEEuwQ6FlLd_mkIfNVzJJk9VgOpkAj9vVyPIvY0p5QJZgz0nDavyeCt_1bhQ-z-l2DPmSWuQt7x4qZ35vFC9ZwMpT7SNIyc0DBbv_Vvv96dM9XsOFlwK5ak-PaEQoDwZuJ9P0eZZ.DR_NR2Ar5Od669JHGxa1wWtJ7iuVQVU3lb1TbZxKfYt_U_DY2y5NM76l32AM-YG8x6Y_f33X8a9G4myIrP-SJFWFgSx_OQSEtLgKfYtVKnv-Bzzzzzzz1I__SZk_LU7BmvUQPOZF8o4pePOQRze-kl-9h9motThHBC0.U1YY0ZDqfykhmfKspynqnfKY5y7_uhD0pyYqnWcd0ATqUvwlnfKdpHdBmy-bIykV0ZKGujYd0APGujYLn0KVIjYknjD4g1DsnHIxnW0dnNt1PW0k0AVG5H00TMfqr0KBpHYkPH9xnW0Yg1RsnsKVm1YknjD4g1DsnHIxnW0dnNts0Z7spyfqn0Kkmv-b5H00ThIYmyTqn0K9mWYsg100ugFM5H00TZ0qn0K8IM0qna3snj0snj0sn0KVIZ0qn0KbuAqs5H00ThCqn0KbugmqTAn0uMfqn0KspjYs0Aq15H00mMTqnH00UMfqn0K1XWY0mgPxpywW5gK1QyIlpZ940A-bm1dcHbD0TA9YXHY0IA7zuvNY5Hm1g1KxnHRs0ZwdT1YknjnYPHbdPjndnjbdnjT4njm3n0Kzug7Y5HDvrjRvrjb1rjfYnW60Tv-b5ymvuWPBPHT1nj0snjDzuHT0mLPV5HbYPjI7rRFDnW0vrHR3wRR0mynqnfKsUWYs0Z7VIjYs0Z7VT1Ys0ZGY5H00UyPxuMFEUHYsg1Kxn7ts0Aw9UMNBuNqsUA78pyw15HKxn7tsg100TA7Ygvu_myTqn0Kbmv-b5H00ugwGujYVnfK9TLKWm1Ys0ZNspy4Wm1Ys0Z7VuWYs0AuWIgfqn0KGTvP_5H00mywhUA7M5HD0UAuW5H00uAPWujY1njPKnWbkPDDkPWD3fRFjrRc3Pj0dPbFDPYn3njuDfsKYTh7buHYs0AFbpyfqfYwAnRc4rRcdf1wDwHm1wW97PjwaPRwAn1nsnjT3rj60mMfqn0KEmgwL5H00ULfqn0KETMKY5H0WnanWnansc10Wna3snj0snj0WnaPDw-fWnanVc108nj0snj0sc1D8nj0snj0sc10WnansQW0snj0sn0KBmy4omyPW5H0Wnansc100TNqv5H08rj-xna3sn7tsQW0sg108rjuxna3dndtsg108PHPxn0KBTdqsThqbpyfqn0KzUv-hUA7M5H00TMw9pyfq0APv5fKGTdqWTADqn0KWTjYs0AN1IjYs0APzm1Ykn16kn6&us=newvui&word=&ck=2654.41.16567.0.0.275.224.0&shh=www.baidu.com&sht=88093251_109_hao_pg&wd=).

**Synthesis of pVIM microgel glues**

Microgels with different EO_2_/VIM ratios were synthesized by free radical precipitation polymerization. In a typical procedure, the total monomer amount was fixed at 6.0 × 10^-3^ mol. EO_2_ and VIM were weighed according to the designed feed ratios of 1:0 (pEO_2_), 2:1 (pVIM-1), and 1:1 (pVIM-2), while the amounts of all other components were kept constant. EO_2_, VIM, PEGDMA (2.0 × 10^-5^ mol, 0.28 g), and SDS (3.6 × 10^-4^ mol, 0.10 g) were dissolved in 200.0 mL of water in a three necked round bottom flask equipped with mechanical stirring, a nitrogen inlet, and a condenser. The solution was purged with nitrogen for 30 min and then heated to 70.0 °C. AAPH initiator solution (1.0 mL, 0.105 mol·L^-1^) was added to start the polymerization, which proceeded for 5 h under continuous stirring. The resulting microgel dispersions were purified by dialysis (molecular weight cutoff 14,000 Da) against water for 3 days at 25.0 °C, with water replaced twice daily. Unless otherwise specified, all experiments involving pVIM microgel glues were conducted using the pVIM-1 formulation.

**Synthesis of Fe_3_O_4_ nanoparticles**

Fe_3_O_4_ NPs were synthesized by mixing Fe^3+^ and Fe^2+^ ions with a 2:1 molar ratio.^1^ Typically, 1.458 g FeCl_3_·6H_2_O (54 mM) and 0.536 g FeCl_2_×4H_2_O (27 mM) were dissolved in 100 mL water at 85 °C with N_2_ gas inlet and vigorous stirring. Then, 2.5 mL of 28% NH_4_OH was added to the reaction mixture dropwise, and the reaction continued for 30 min. After cooling down to room temperature, the resulting black precipitates were magnetically separated and washed multiple times with water. The mass concentration of Fe_3_O_4_ NPs was 6.25 mg⋅mL^-1^.

**Synthesis of Fe_3_O_4_@pVIM microgel glues**

For the fabrication of hybrid microgels, 50 mL of pure pVIM microgel dispersion and 50 mL ultrapure water was taken in a three necked round bottom flask with N_2_ gas inlet and stirring using magnetic stirrer. 1.458 g FeCl_3_×6H_2_O (54 mM) and 0.536 g FeCl_2_×4H_2_O (27 mM) solution was added and stirring was continued for 30 min. After N_2_ purging for 30 min, the temperature was increased to 85.0 °C, then NH_4_OH (2.5 mL, 28%) was added to reaction mixture and again stirring was continued for 30 min with N_2_ gas inlet. After cooling down to room temperature, the resulting black precipitates were magnetically separated and washed multiple times with water. The mass concentration of Fe_3_O_4_@pVIM was 8.52 mg⋅mL^-1^.

**Preparation and characterization of microplastic particles**

50 nm, 100 nm, 500 nm, and 1 μm monodisperse fluorescent polystyrene microspheres (green emission, 488/520 nm; 25 mg⋅mL^-1^; 10 mL dispersion) were purchased from Zhichuan Microsphere Bioscience Co., Ltd. (Jiangsu, China). The product was supplied as a uniform spherical particle dispersion and used as received. The morphology were further confirmed by TEM (Figure S4).

Polystyrene (PS), polyethylene terephthalate (PET), polypropylene (PP), and polyethylene (PE) were obtained from common commercial products (PS cutlery, PET bottles, PP containers, and PE films). The bulk plastics were first trimmed into small pieces and then pre-fragmented using a high-speed rotary blade grinder (~20,000 rpm). To further reduce the particle size and obtain microplastics, the resulting fragments were subjected to cryogenic milling in liquid nitrogen (-196 ^o^C) for 30 min and subsequently passed through stainless-steel sieves to isolate particles in the 50~100 μm range. The microplastics (50~100 μm) were then collected and dried at room temperature under vacuum. SEM imaging confirmed the irregular flake-type structures of mechanically fragmented PS, PET, PP, and PE (Figure S5). FTIR spectra were used to confirm polymer identity, showing characteristic aromatic (PS), ester (PET), and alkyl stretching signals (PP and PE) (Figure S6). *ζ*-Potential measurements in ultrapure water (pH =6.5.0) revealed negative surface charges for all microplastics, consistent with surface oxidation introduced during commercial processing or fragmentation (Figure S7).

**Preparation of microplastic dispersions**

Microplastic stock dispersions were prepared by accurately weighing the required amount of PS, PET, PP, or PE particles (50~100 μm) and dispersing them in ultrapure water (18.2 MΩ·cm, 25 ^o^C). The dispersions were then subjected to continuous agitation on an orbital shaker (SK-O180-S) at 200 rpm for 24 h to ensure complete wetting and uniform dispersion. Owing to their small particle size and low concentration, the resulting microplastic dispersions remained stably suspended without visible sedimentation; long-term monitoring of the supernatant using fluorescence spectroscopy (F-7000) confirmed that the dispersions remained homogeneous for at least three months under quiescent storage conditions. Environmental matrices were also examined by replacing ultrapure water with natural lake water (collected from Furong Lake at Xiamen University) or seawater (sampled from Baicheng Beach near the Xiamen University coastline), following the same dispersion and mixing procedures.

**Preparation of the standard calibration curve for fluorescent PS microspheres (50 nm~1 μm)**

To establish a standard calibration curve, a series of PS solutions with concentrations of 0.01, 0.02, 0.03, 0.04, 0.05, 0.1, and 0.2 mg⋅mL^-1^ were prepared by diluting the PS stock solution (25.0 mg⋅mL^-1^) with ultrapure water (18.2 MΩ·cm). The fluorescence intensity of these solutions was measured using F-7000 Molecular Fluorescence Spectrometer under the following conditions: excitation wavelength of 488 nm, emission wavelength of 520 nm, and a slit width of 5 nm. Ultrapure water was used as a blank control. The resulting fluorescence intensities were plotted against the corresponding MPs concentrations to construct the standard calibration curve.

**Characterization and methods**

**Removal of 50 nm~1 μm fluorescent PS microspheres**

For the removal experiment, a PS stock solution (25.0 mg⋅mL^-1^) was diluted with ultrapure water to the desired concentration in a total volume of 20 mL. A specified volume of the pVIM microgel glues’ dispersion was added to the corresponding sample vial using a pipette. The mixture was placed on an orbital shaker (SK-O180-S) at 200 rpm at 25 °C for 60 min to ensure thorough interaction between the PS microspheres and pVIM microgel glues.

The effect pVIM dosage (0.1, 0.15, 0.2, 0.3, 0.4, 0.5, 0.8 and 1.0 mg⋅mL^-1^) on the removal efficiency of fluorescent PS microspheres was investigated under experimental conditions with an initial PS concentration of 1 mg⋅mL^-1^.

For kinetic studies, samples were withdrawn at designated time points. After mixing pVIM (0.2 mg⋅mL^-1^) and PS MPs (1.0 mg⋅mL^-1^), the system (100.0 mL) shaken on an orbital shaker at 200 rpm for 120 min. A pipette was used to collect the supernatant (0.2 mL) from 2 cm below the liquid surface for fluorescence intensity measurements to quantify the residual PS microspheres. The collected aliquot was then diluted tenfold with ultrapure water prior to fluorescence measurements, which were used to quantify the residual PS microspheres. Each experiment was performed in triplicate, and a blank control was included for comparison.

The removal of PS microspheres was evaluated using both the removal efficiency (*RE*) and the adsorption capacity (*q*), calculated according to Equations (1) and (2), respectively:

$\begin{aligned} \text{RE}\text{=}\frac{\text{C}_{\text{0}}\text{-}\text{C}_{\text{t}}}{\text{C}_{\text{0}}}\text{ × 100\% }\left（ \text{1} \right） \end{aligned}$

$\begin{aligned} \text{q}\text{=}\frac{{\text{(}\text{C}}_{\text{0}}\text{-}\text{C}_{\text{t}}\text{)}\text{V}}{\text{m}}\text{ × 100\% }\left（ \text{2} \right） \end{aligned}$

Where *C*_0_ and *C_t_* (mg⋅mL^-1^) represent the initial and final concentrations of PS microspheres in the solution, respectively; m (g) is the mass of pVIM added; and *V* (mL) is the volume of the solution.

**Magnetic removal of 50~100 μm microplastics**

Typically, Fe_3_O_4_@pVIM microgel glues (1 mg⋅mL^-1^) were mixed with microplastic dispersions (4 mg⋅mL^-1^), and the mixtures were gently agitated on an orbital shaker (SK-O180-S) at 200 rpm. After 60 min of adhesion-driven aggregation, the microplastics were magnetically separated from the suspension by applying an external magnet. The collected solids were subsequently dried under vacuum, weighed, and the microplastic removal ratio was calculated.

**CO_2_ response performance test**

The pure CO_2_ and N_2_ gas were collected in balloons and introduced into the diluted microgel dispersion to evaluate the responsibility of the microgels. Each gas is bubbled for 20 min. DLS is used to detect the hydrodynamic diameter (<*D*>_h_) of microgels, the zeta-potential (*ζ* potential) and pH are used to detect the charge state of microgels.

**Desorption Studies**

To investigate the interaction and desorption behavior between Fe_3_O_4_@pVIM microgels and PS MPs, desorption study was conducted. Initially, 0.3 mg⋅mL^-1^ Fe_3_O_4_@pVIM microgels were incubated with 100 nm PS microspheres (1.0 mg⋅mL^-1^) for 60 min to allow for sufficient adsorption. After adsorption, the supernatant was carefully removed to eliminate unbound microgels. The same volume of deionized water was then added to maintain the original suspension volume. N_2_ was subsequently bubbled into the system, followed by shaking on a thermostatic orbital shaker at ambient temperature for 60 min. Desorption process of Fe_3_O_4_@pVIM was visually tracked by the appearance of a yellow coloration in the supernatant, indicating the release of PS microspheres. Quantitative analysis was performed by measuring the fluorescence intensity of the collected supernatant.

**Other Characterization**

The X-ray photoelectron spectroscopy (XPS, PHI Quantum-2000) experiment was carried out using a custom-built five-chamber ultrahigh vacuum system, with the base pressure lower than 5x10-11Torr. The XPS spectrometer consisted of a SPHERA hemispherical electron analyzer with a monochromatic Al Ka (1486.7 eV) radiation source. X-ray diffraction (XRD) analysis was performed using a Rigaku Ultima IV diffractometer (Japan) operating at 40 kV and 30 mA. The samples were placed on a quartz substrate and scanned at a rate of 10° min⁻¹ for phase identification. The ζ-potential and hydrodynamic size were measured by a dynamic light scattering (DLS) instrument, A standard laser light scattering spectrometer (BI-200SM) equipped with a BI-9000 AT digital time correlator (Brookhaven Instruments, Inc.), a Mini-L30 diode laser (30 mW, 637 nm) as the light source and a temperature controller (±0.1 °C) was used. The vibrating sample magnetometry (VSM, Lake Shore Cryotronics, Inc.) measurements were run on a Quantum Design Magnetic Properties Measurement System(QD MPMS-XL) at a temperature of 300K with an applied field ranging from +/- 50 kOe. Transmission electron microscopy (TEM) images were acquired on a JEOL JEM 1400 operating at an acceleration voltage of 100 kV. Samples were prepared by drying a 10 μL drop of colloidal dispersion on a carbon-coated copper grid. IR spectra were recorded on a Thermo Electron Corporation Nicolet 380 Fourier transform infrared spectrometer. NMR spectra were recorded on a Bruker AVIII 400 MHz solution-state NMR spectrometer. Differential scanning calorimetry (DSC) measurements were performed on a NETZSCH DSC 204 F1 Phoenix system under a nitrogen atmosphere with a flow rate of 40 mL⋅min⁻¹. Samples (5-10 mg) were sealed in aluminum pans and subjected to a heating-cooling-heating cycle at a rate of 10 K⋅min⁻¹ over the temperature range of -40 °C to 20 °C. The glass transition temperature (Tg) was determined from the second heating scan to eliminate thermal history effects, with the Tg value taken as the midpoint of the step-like transition in the heat flow curve, and confirmed by the minimum of the first derivative of the heat flow (dHF/dT) curve.

**Figures and Tables**


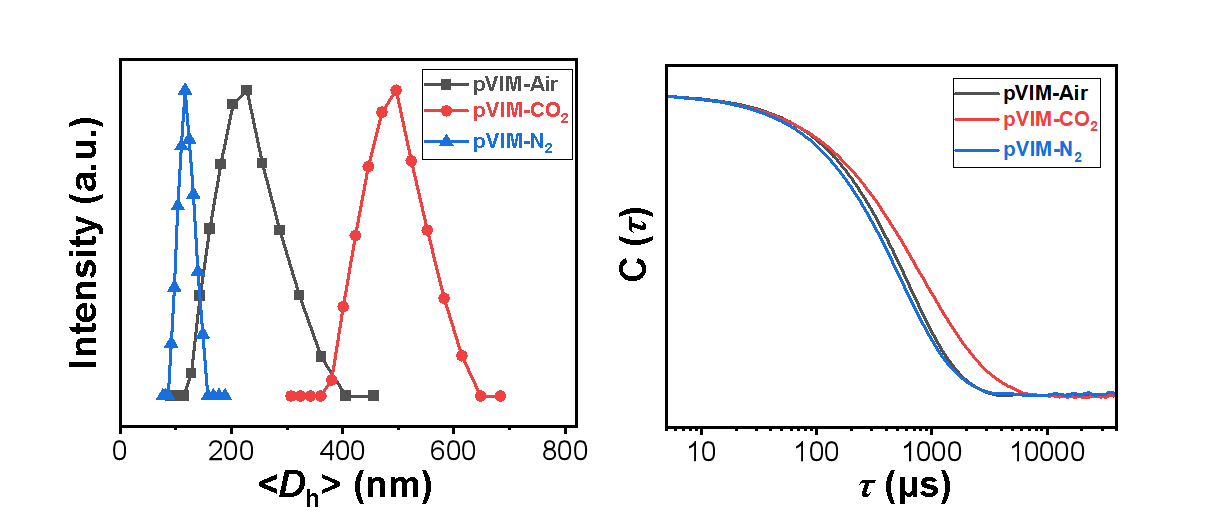


**Figure S1.** DLS size distribution and intensity autocorrelation function of pVIM microgel glues under air, CO_2_, and N_2_ atmospheres.


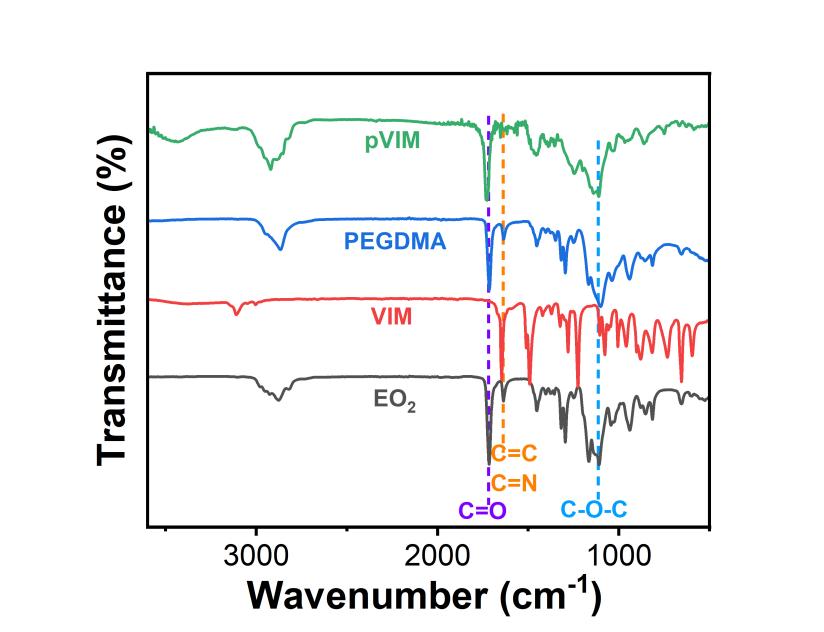


**Figure S2.** A comparison of the FTIR spectra of pVIM microgel glues, EO_2_ monomer, VIM monomer,and PEGDMA monomer.


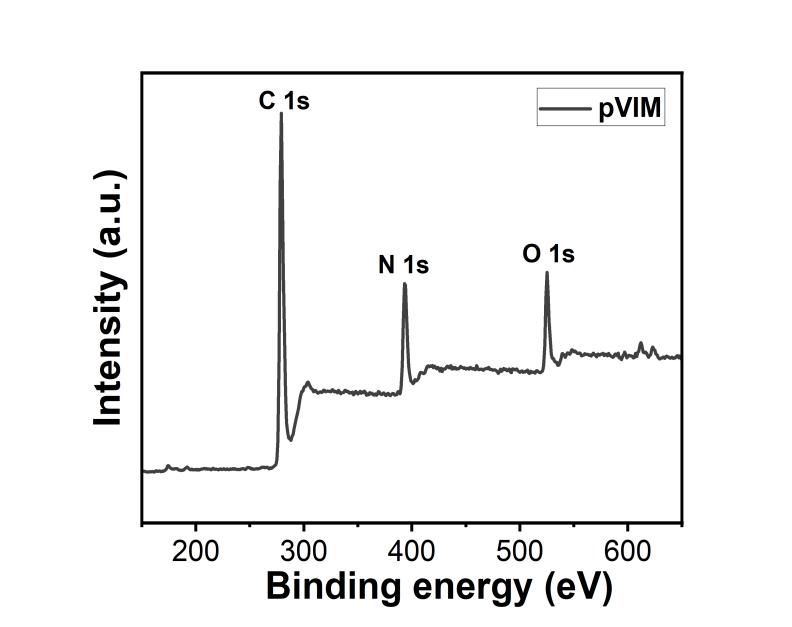


**Figure S3.** XPS spectra of pVIM microgel glues showing the characteristic signals of C 1s, N 1s, and O 1s.


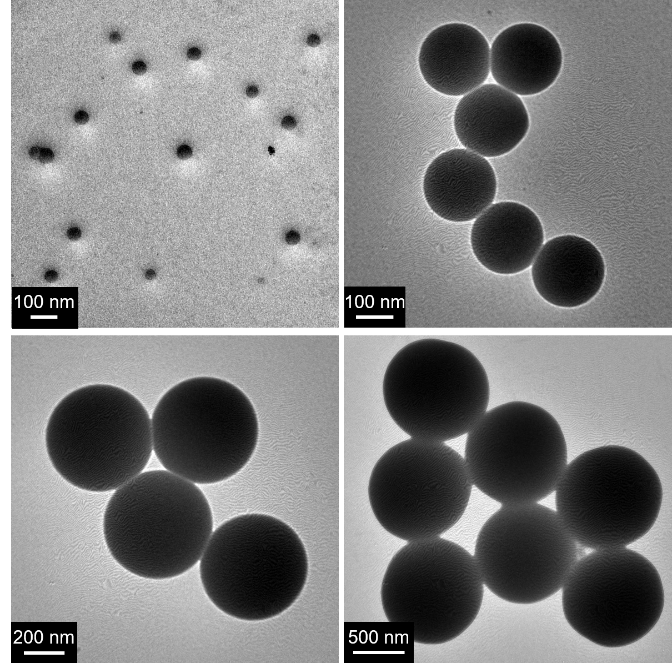


**Figure S4.** TEM images of 50 nm, 200 nm, 500 nm and 1 μm PS fluorescent microspheres.


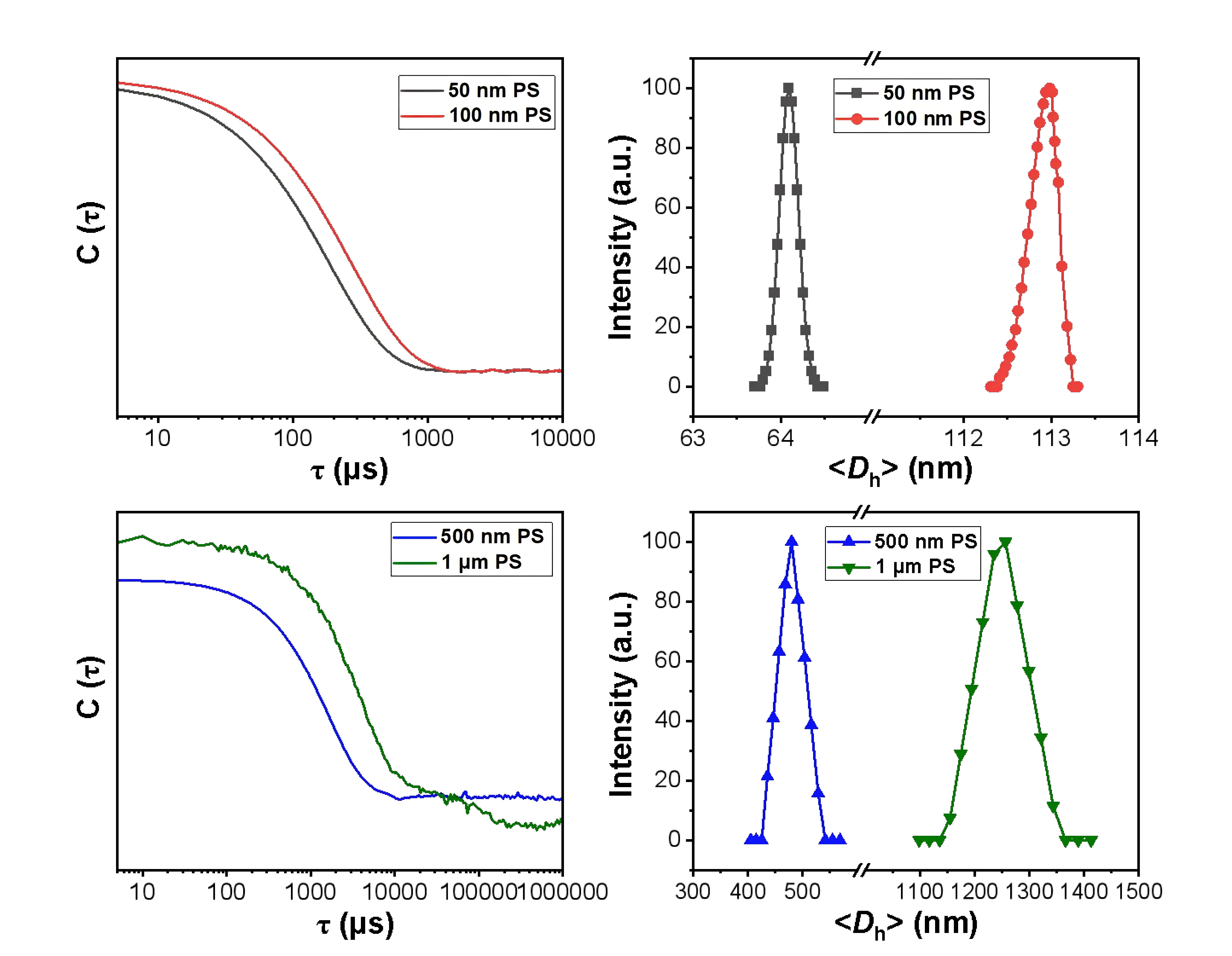


**Figure S5.** Intensity autocorrelation function and DLS size distribution of 50 nm, 100 nm, 500 nm and 1 μm PS fluorescent microspheres.


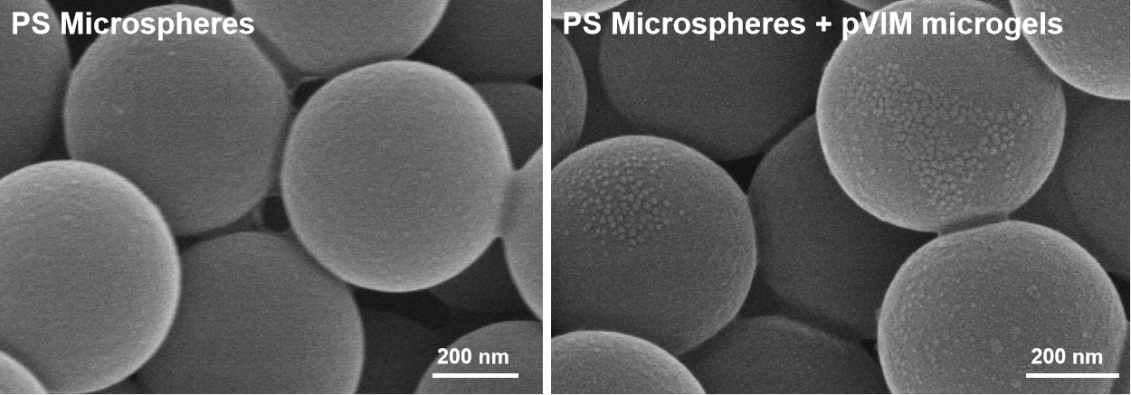


**Figure S6.** SEM images of pristine PS microspheres (500 nm) and PS microspheres after incubation with pVIM microgel glues, clearly revealing the presence of adhered spherical microgel particles distributed on the PS surface.


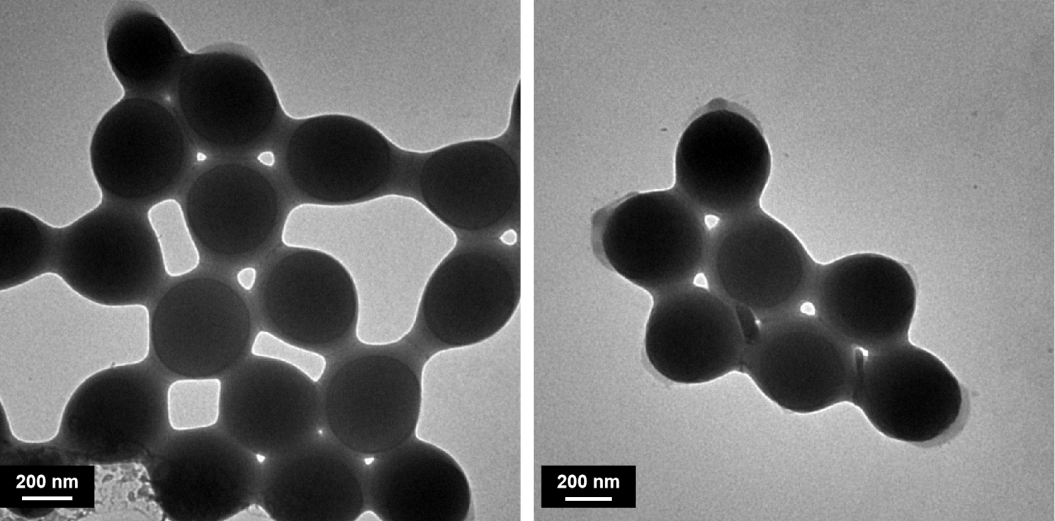


**Figure S7.** TEM images of PS microspheres (500 nm, 1.0 mg⋅mL^-1^) after incubation with pVIM microgel glues (0.2 mg⋅mL^-1^), showing a distinct low-contrast adhesive layer surrounding the particle surface.


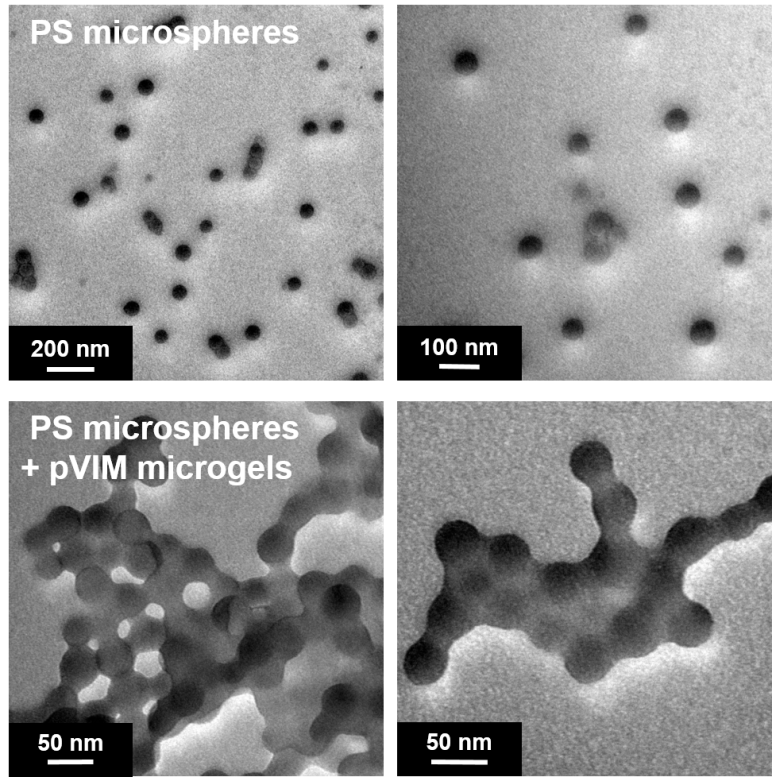


**Figure S8**. TEM images of PS microspheres (50 nm, 1.0 mg⋅mL^-1^) and PS microspheres after incubation with pVIM microgel glues (0.2 mg⋅mL^-1^), showing a distinct low-contrast adhesive layer surrounding the particle surface.


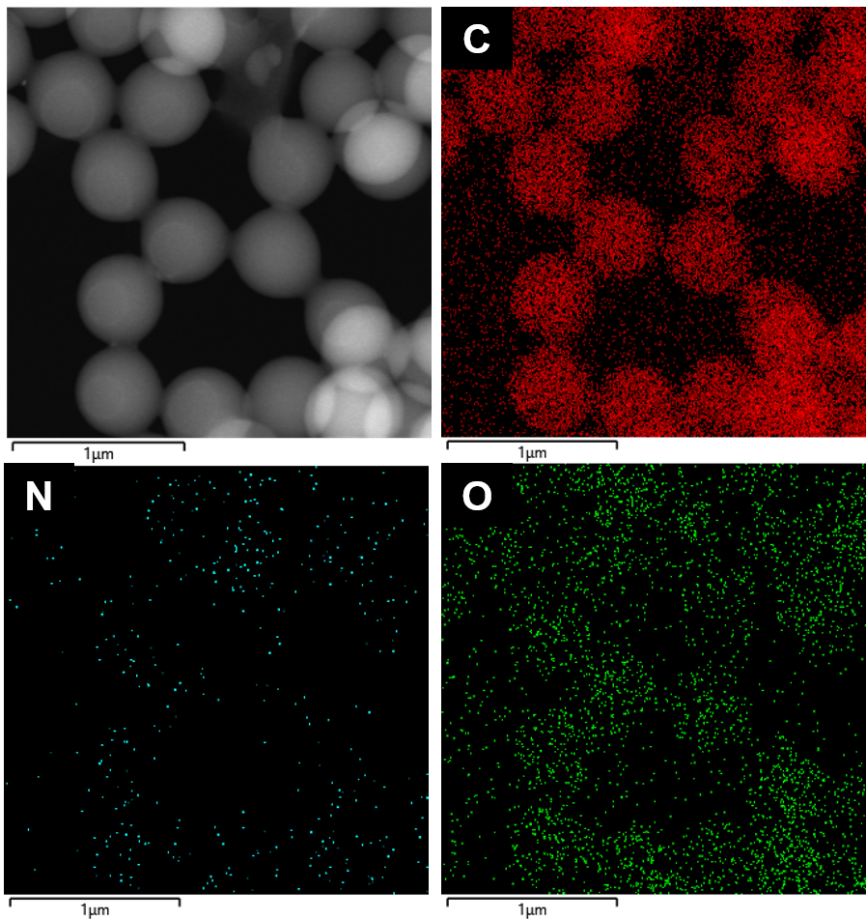


**Figure S9.** TEM-EDS elemental mapping images of PS microspheres (500 nm) after incubation with pVIM microgel glues.


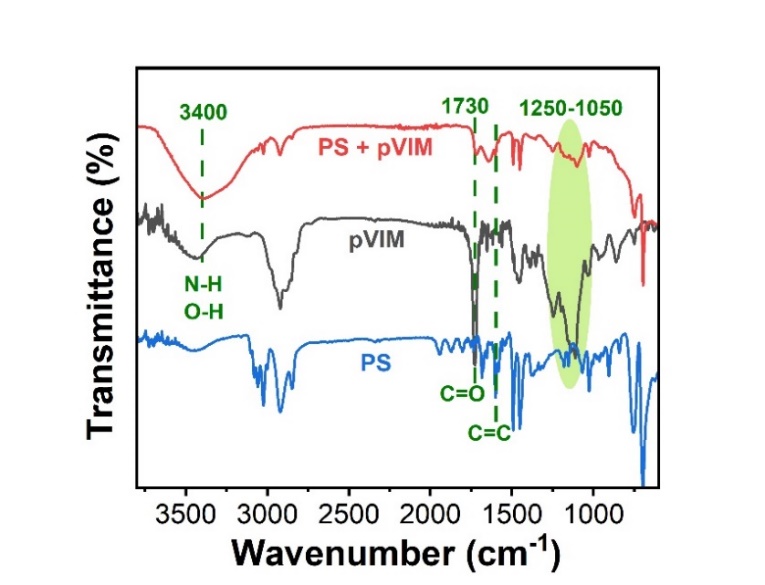


**Figure S10.** ATR-FTIR spectra of PS, pVIM, and PS-pVIM composites, showing characteristic peak shifts that confirm interfacial interactions between the pVIM microgel glues and PS microspheres.


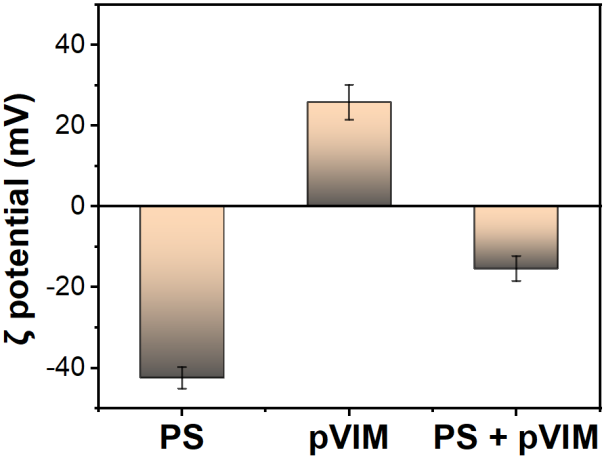


**Figure S11.** Zeta potential of PS microspheres (500 nm), pVIM microgel glues and PS-pVIM composites.


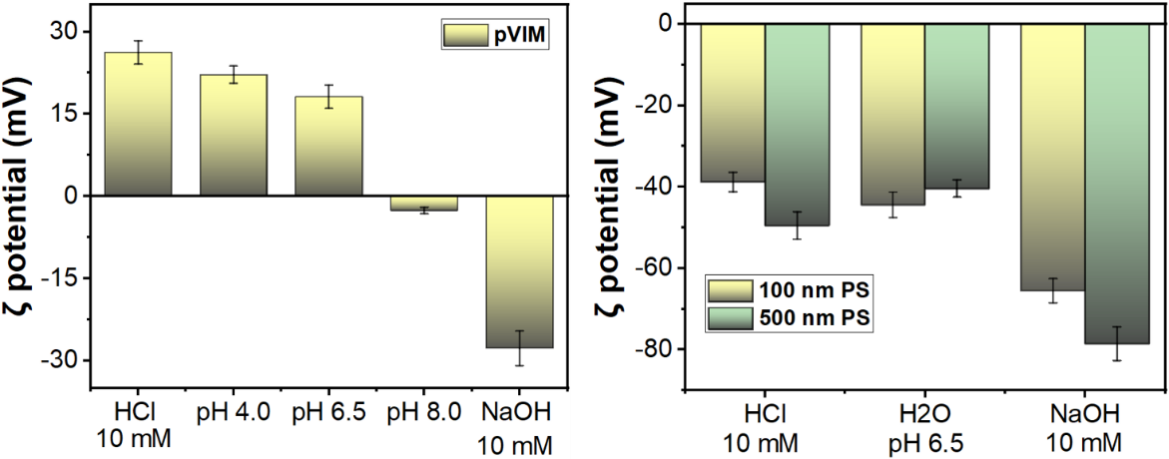


**Figure S12.** Zeta potential measurements of pVIM microgel glues and PS microspheres (100 nm and 500 nm) under different pH conditions.


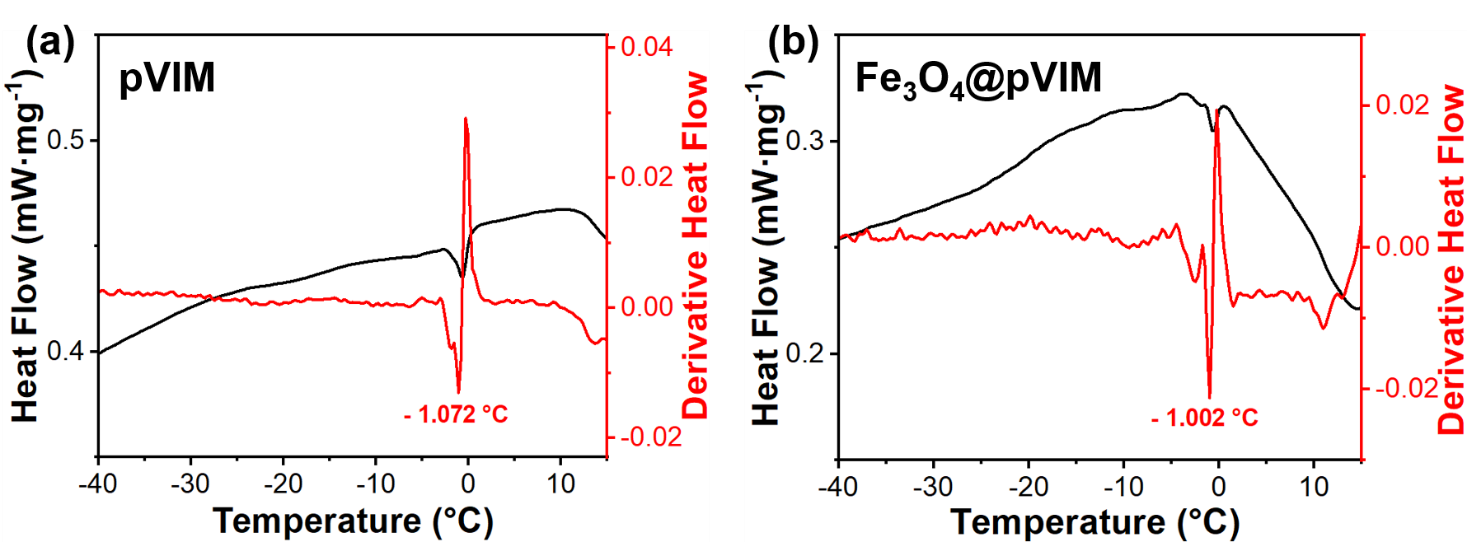

**Figure S13**. DSC curves and glass transition temperatures of pVIM microgel glues (a) and Fe₃O₄@pVIM composite microgel glues (b).


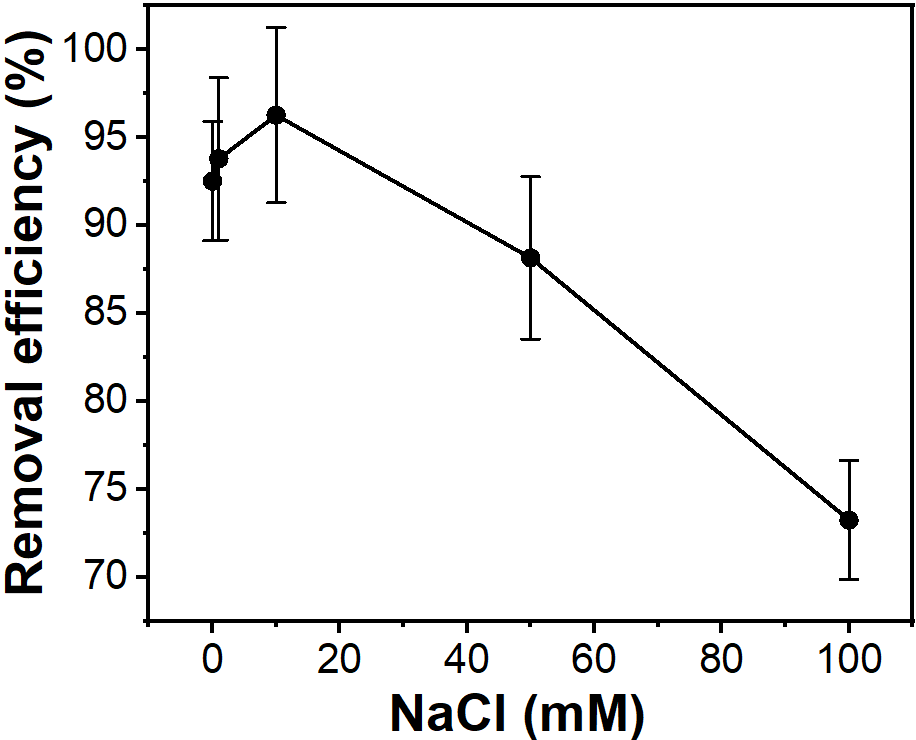


**Figure S14.** Effect of NaCl concentration on the removal efficiency of PS microspheres by pVIM microgel glues.


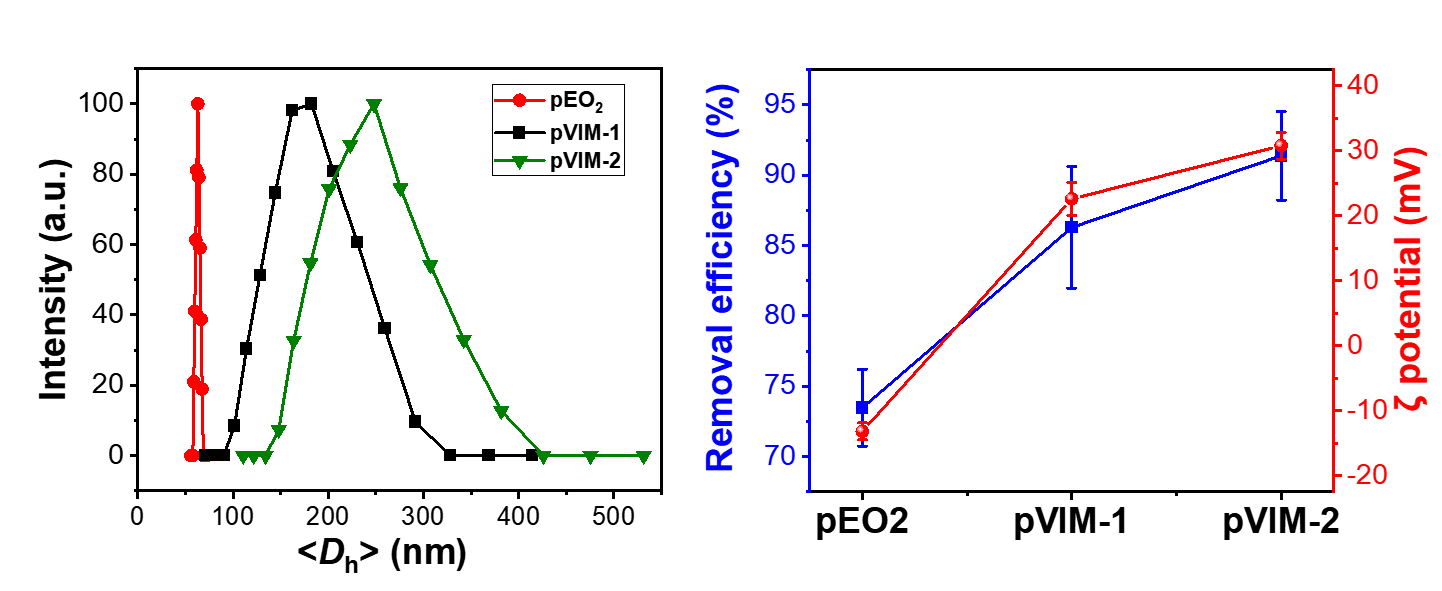


**Figure S15.** DLS size distributions of pEO_2_, pVIM-1 and pVIM-2 microgels, together with their corresponding ζ-potentials and removal efficiencies toward 50 nm PS microspheres.


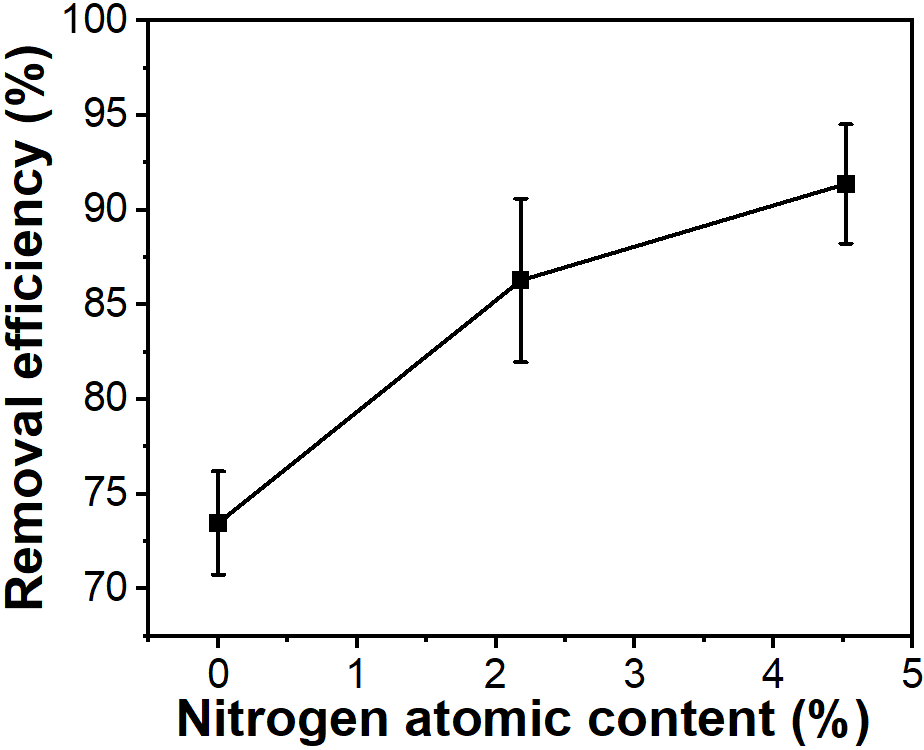


**Figure S16.** XPS-determined surface nitrogen atomic content of pVIM microgels and its correlation with removal efficiency for 50 nm PS microspheres.


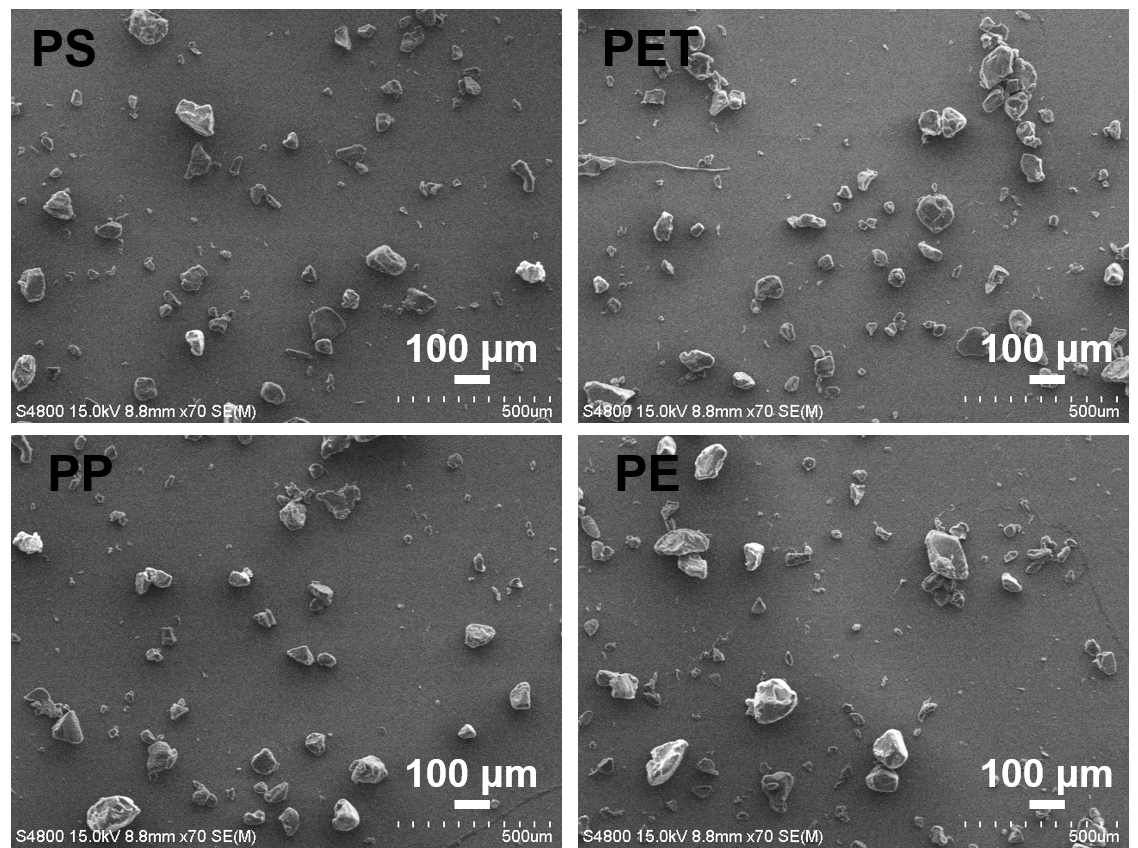


**Figure S17.** SEM images of mechanically fragmented PS, PET, PP and PE particles.


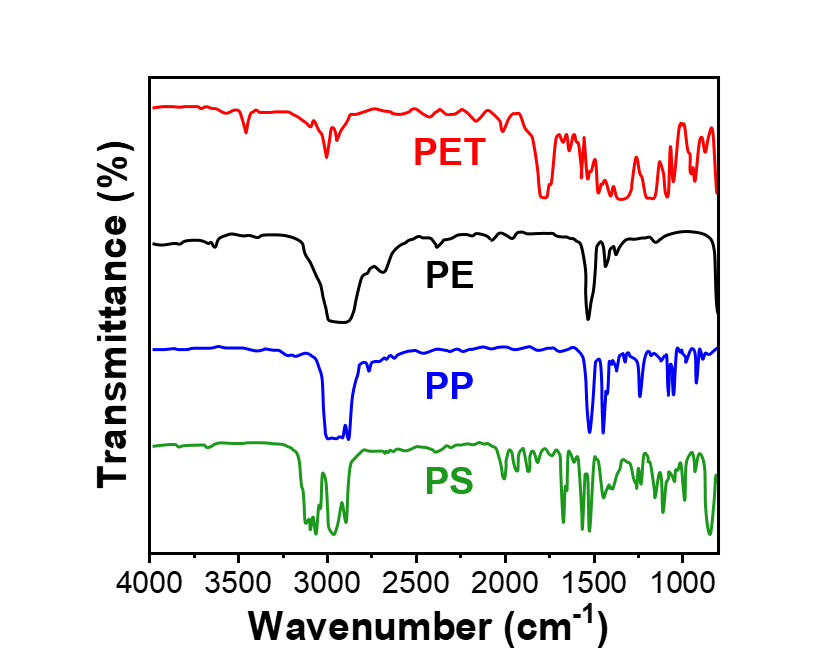


**Figure S18.** FTIR spectra of the four representative microplastic particles (50~100 μm) used in this study, including PS, PET, PP, and PE.


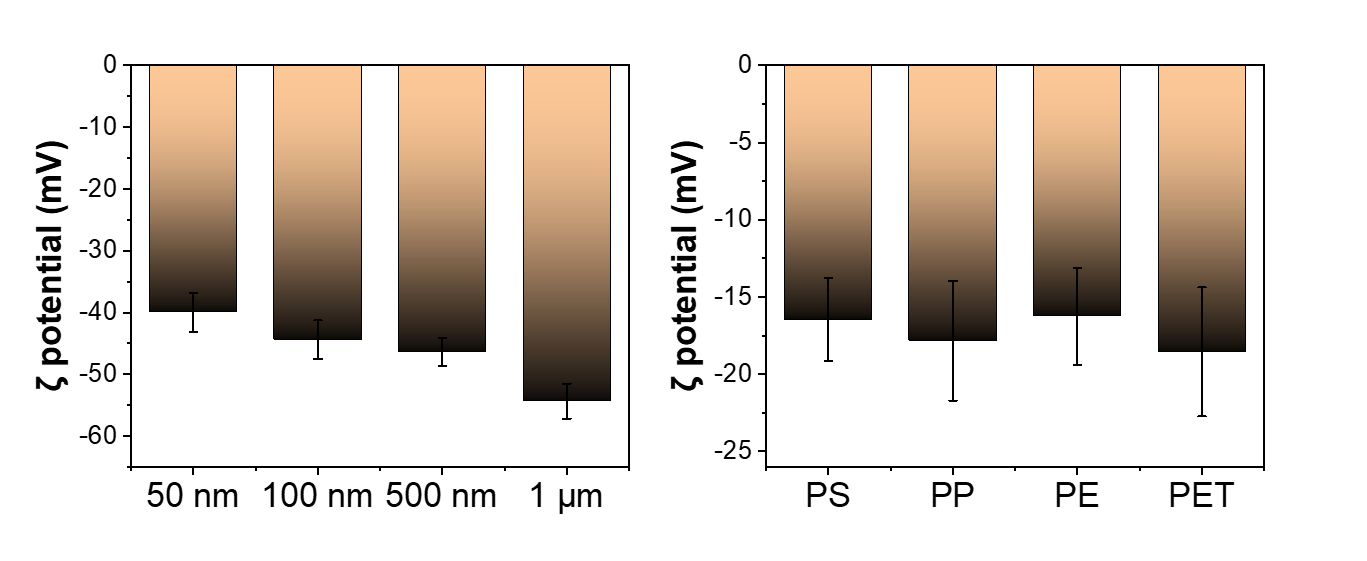


**Figure S19.** Zeta potentials of 50 nm, 100 nm, 500 nm, 1 μm PS fluorescent microspheres and the four representative microplastic particles (PS, PET, PP, and PE, 50~100 μm) measured in ultrapure water.


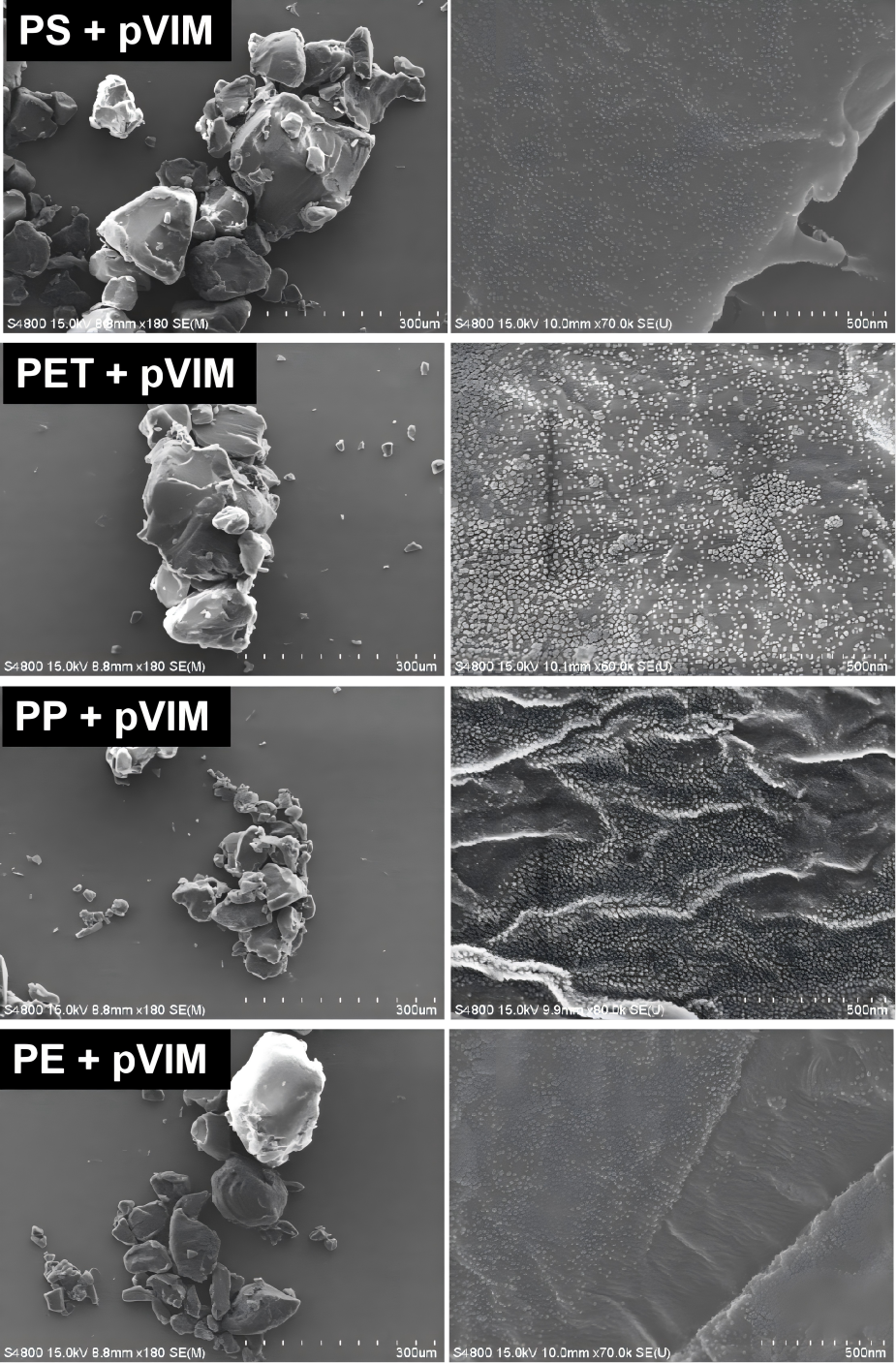


**Figure S20.** A SEM image depicting pVIM microgel glues adhered to the surface of 50-100 μm PS, PP, PE, and PET MPs.


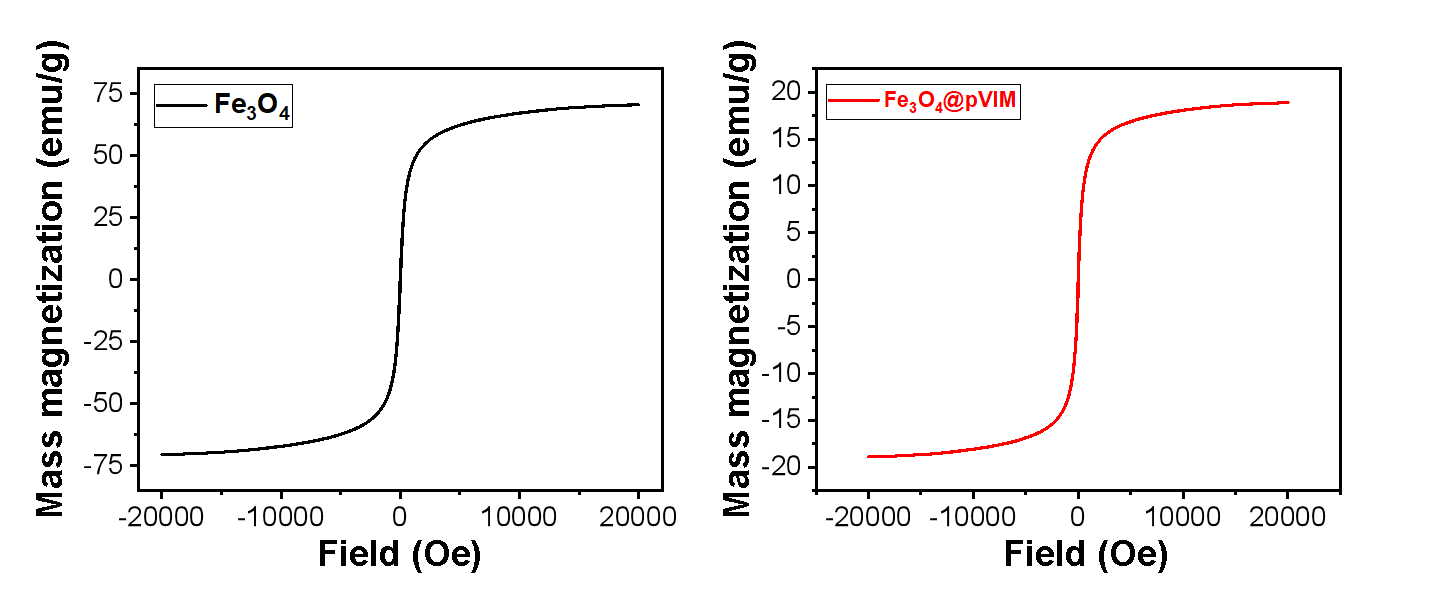


**Figure S21.** VSM magnetization curves of the Fe_3_O_4_ nanoparticles and Fe_3_O_4_@pVIM magnetic microgels.


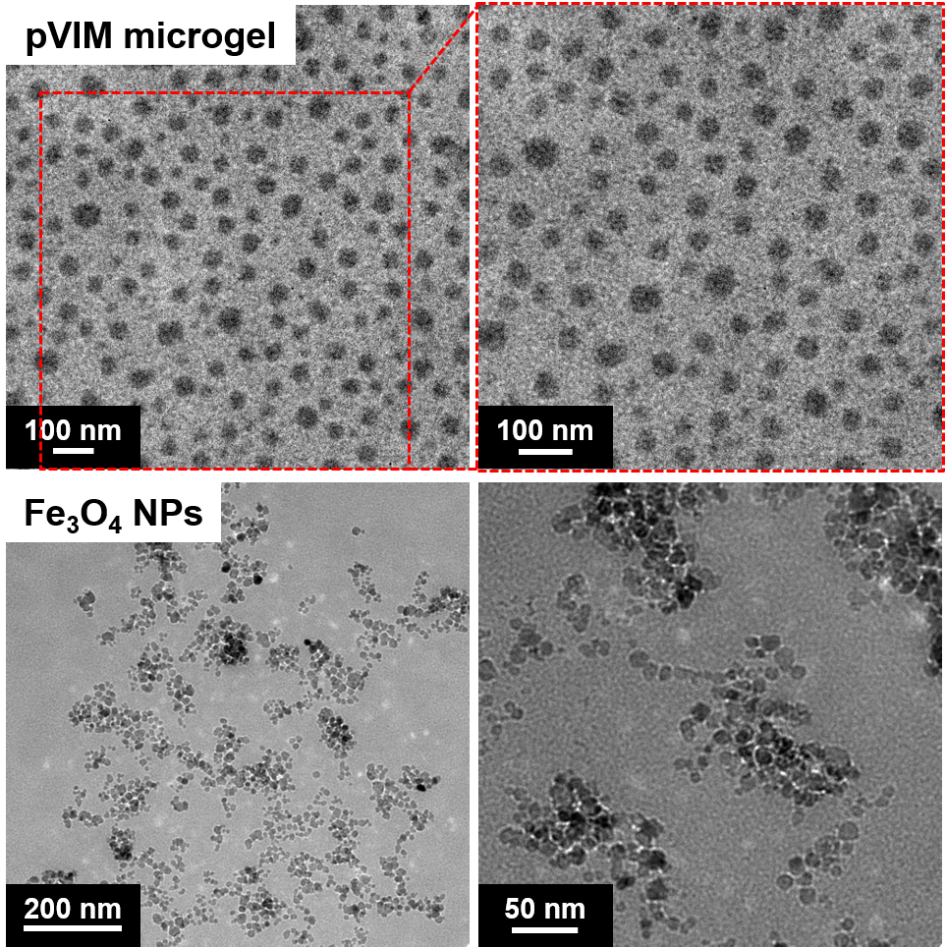


**Figure S22.** TEM images at different magnifications of pVIM microgel glues and Fe_3_O_4_ nanoparticles.


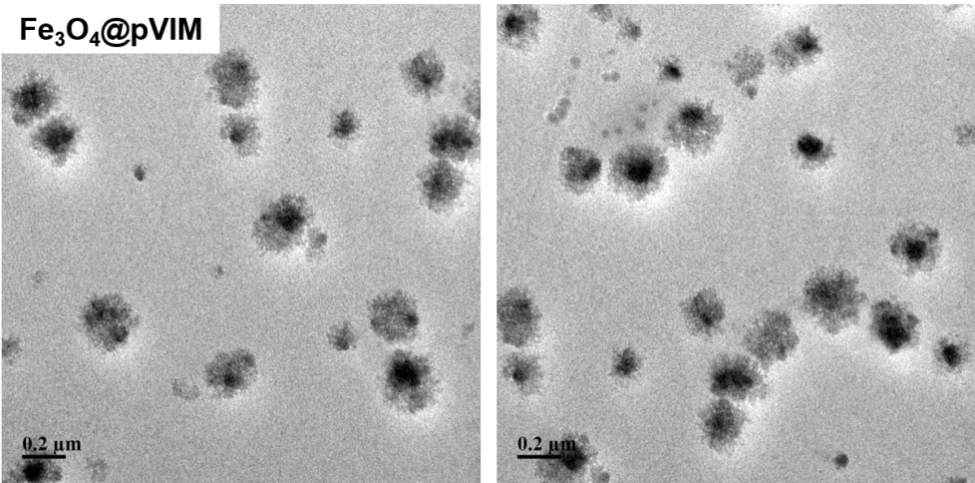


**Figure S23.** TEM images of Fe_3_O_4_@pVIM microgel glues.


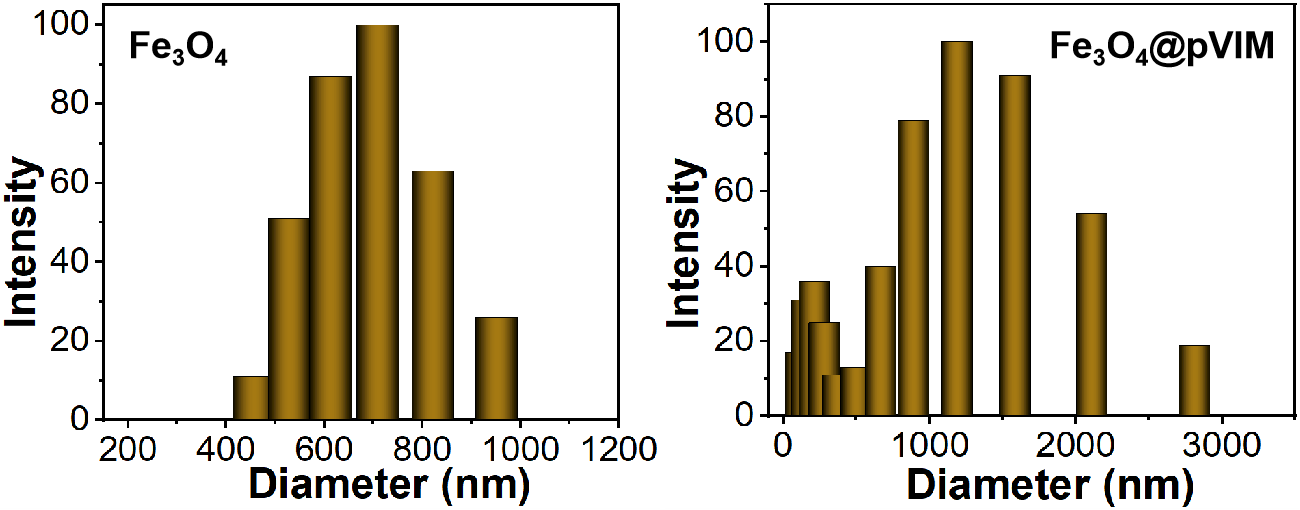


**Figure S24.** DLS size distribution of the Fe_3_O_4_ nanoparticles and Fe_3_O_4_@pVIM microgel glues.


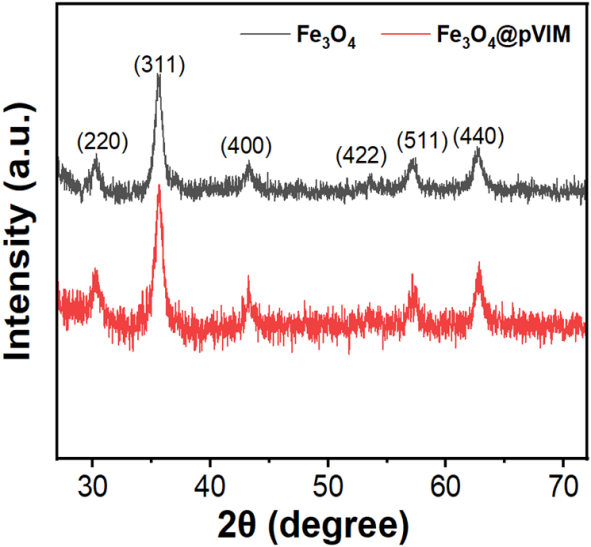


**Figure S25.** XRD spectra of Fe_3_O_4_ nanoparticles and Fe_3_O_4_@pVIM microgel glues.


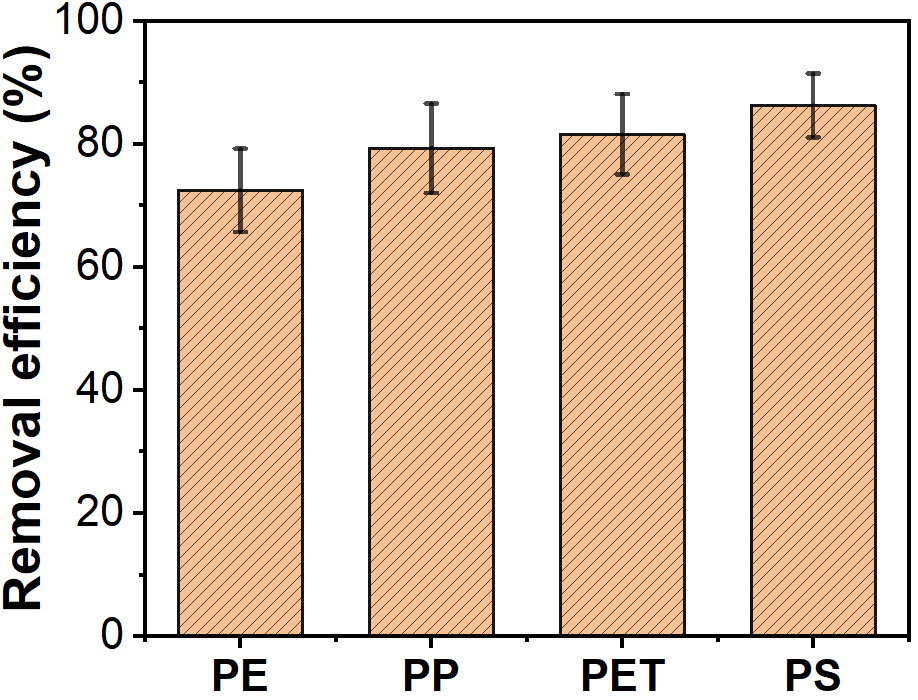


**Figure S26.** Removal efficiencies of different microplastic types (PE, PP, PET, PS; 50-100 μm; the MPs are representative of debris originating from the fragmentation of various commonly used plastic goods) using Fe_3_O_4_@pVIM microgel glues.


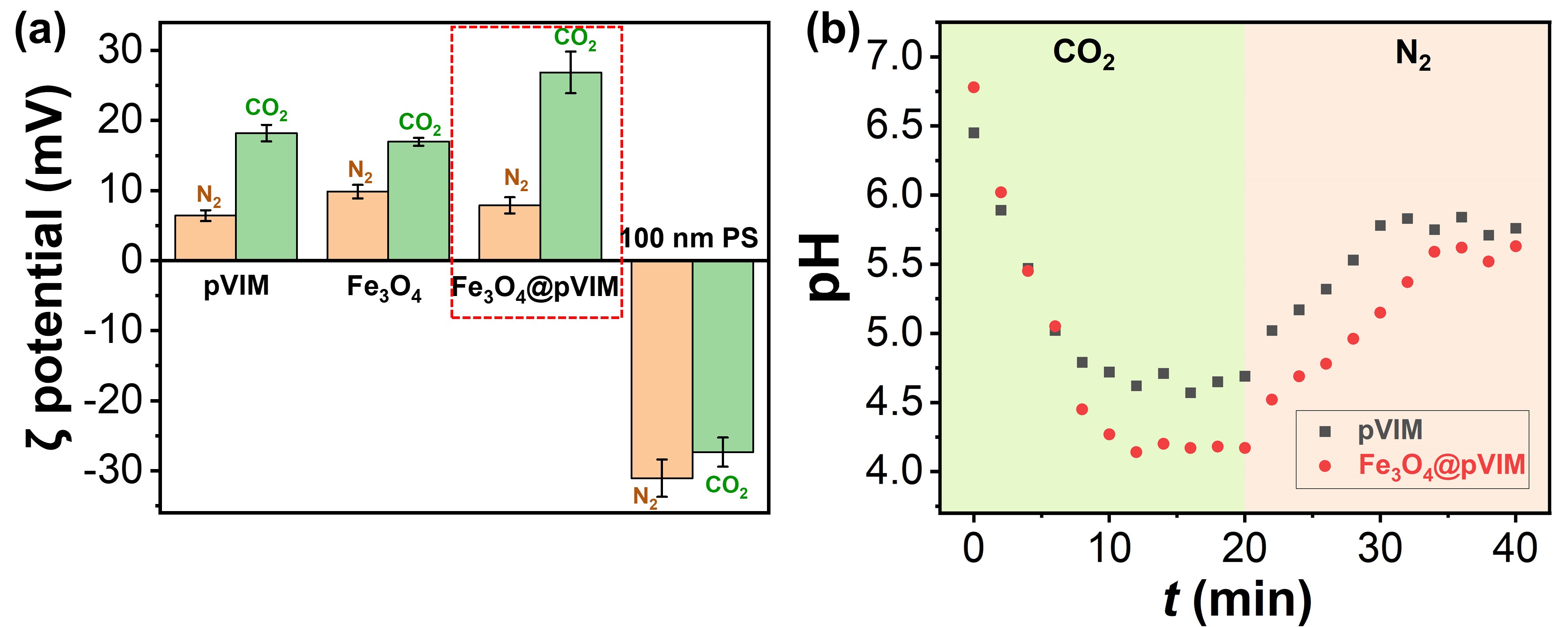


**Figure S27.** (a) ζ-potential of pVIM, Fe_3_O_4_, Fe_3_O_4_@pVIM and 100 nm PS microspheres at the atmosphere of N_2_ and CO_2_. (b) The pH of the pVIM and Fe_3_O_4_@pVIM systems over time as pure CO_2_ and N_2_ were alternately introduced into the microgel dispersion.


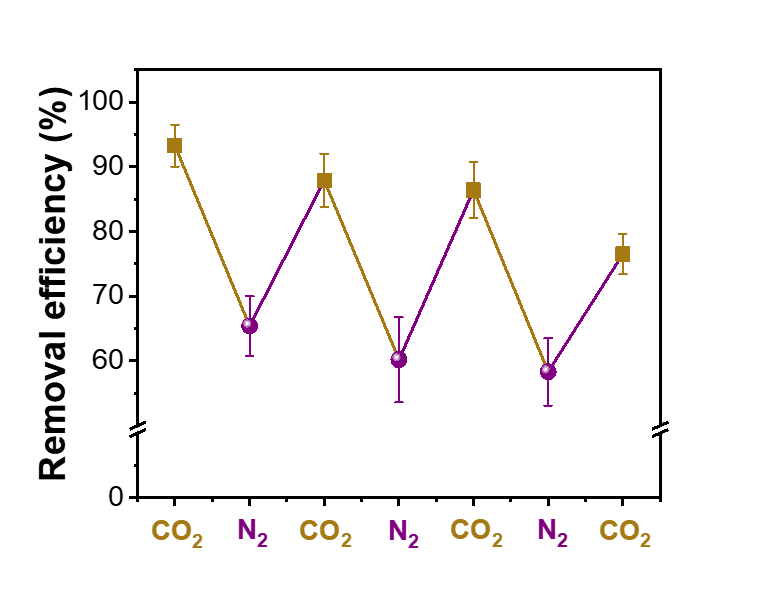


**Figure S28.** Reversible adsorption-desorption behavior of 100 nm PS microspheres upon alternating CO_2_/N_2_ bubbling cycles.

**Table S1.** Kinetic parameters and correlation coefficients obtained from pseudo first order and pseudo second order fitting models for PS microspheres removal by Fe_3_O_4_@pVIM microgel glues

| **Sample** | **Pseudo-first-order** | | | **Pseudo-second-order** | | |
| --- | --- | --- | --- | --- | --- | --- |
|  | *k*_1_  (min^-1^) | *q*_e,cal_  (mg⋅g^-1^) | *R*² | *k*_2_  (g⋅ mg^-1^⋅min^-1^) | *q*_e,cal_  (mg⋅g^-1^) | *R*² |
| 100 nm PS | 0.0148 | 1995.5 | 0.94 | 2.3 × 10^-5^ | 1890.0 | 0.98 |
| 500 nm PS | 0.0185 | 1734.8 | 0.99 | 1.8 × 10^-5^ | 1880.7 | 0.98 |
| 1 μm PS | 0.0442 | 1417.0 | 0.99 | 3.9 × 10^-5^ | 1499.5 | 0.97 |

**Table S2.** Comparison of microplastic removal efficiency between this work and a few literature reported methods.

| **Reference** | **Magnetic NPs concentration** | **Microplastic concentration** | **Magnetic NP to microplastics ratio (w/w)** | **Microplastics type and size** |
| --- | --- | --- | --- | --- |
| [1] | 40 μg/mL | 4 mg/mL PS | 1:100 | fragments <1 mm |
| [2] | 10 mg/mL | 1 mg/mL PS | 10:1 | beads 1-10 μm |
| [3] | 0.1% wt | 0.1% wt PS | 1:1 | beads <10 μm |
| [4] | 2 mg/mL | 2 mg/mL PP | 1:1 | fragments <50 μm |
| This work | 0.3 mg/mL | 1 mg/mL PS | 1: 3 | beads ≤1 μm fragments 50-100μm |

**Table S3.** Physicochemical parameters of tested natural waters.

| **Parameter** | **Ultrapure Water** | **Lake Water (from Furong Lake at Xiamen University)** | **Seawater (from Baicheng Beach near Xiamen University)** |
| --- | --- | --- | --- |
| pH | 6.8 | 7.1 | 8.1 |
| Turbidity (NTU) | <0.1 | ~5.5 | ~2.7 |
| Conductivity (μS⋅cm^-1^) | <1 | ~346 | ~45479 |
| Total Dissolved Solids, TDS (mg⋅L^-1^) | <1 | ~218 | ~34227 |

**References for Supporting Information**

1. Zandieh, M.; Liu, J., Removal and Degradation of Microplastics Using the Magnetic and Nanozyme Activities of Bare Iron Oxide Nanoaggregates. *Angewandte Chemie International Edition* **2022,** *61* (47).

2. Misra, A.; Zambrzycki, C.; Kloker, G.; Kotyrba, A.; Anjass, M. H.; Franco Castillo, I.; Mitchell, S. G.; Guttel, R.; Streb, C., Water Purification and Microplastics Removal Using Magnetic Polyoxometalate-Supported Ionic Liquid Phases (magPOM-SILPs). *Angew Chem Int Ed Engl* **2020,** *59* (4), 1601-1605.

3. Sarcletti, M.; Park, H.; Wirth, J.; Englisch, S.; Eigen, A.; Drobek, D.; Vivod, D.; Friedrich, B.; Tietze, R.; Alexiou, C.; Zahn, D.; Apeleo Zubiri, B.; Spiecker, E.; Halik, M., The remediation of nano-/microplastics from water. *Materials Today* **2021,** *48*, 38-46.

4. Zhou, H.; Mayorga-Martinez, C. C.; Pumera, M., Microplastic Removal and Degradation by Mussel-Inspired Adhesive Magnetic/Enzymatic Microrobots. *Small Methods* **2021,** *5* (9), e2100230.
